# Latent tuberculosis infection in focus: Evaluating public concerns, treatment perceptions, and awareness in King Sabatha Dalindyebo Local Municipality, Eastern Cape, South Africa

**DOI:** 10.1371/journal.pgph.0005130

**Published:** 2026-09-15

**Authors:** Lindiwe Modest Faye, Ncomeka Sineke, Ntandazo Dlatu, Urgent Tsuro, Mojisola Clara Hosu, Teke Apalata

**Affiliations:** 1 School of Pathology, Faculty of Medicine and Health Sciences, Walter Sisulu University, Mthatha, South Africa; 2 School of Public Health, Walter Sisulu Institute for Clinical Governance and Healthcare Administration, Faculty of Health Sciences, Walter Sisulu University, Mthatha, South Africa; PLOS: Public Library of Science, UNITED STATES OF AMERICA

## Abstract

Latent tuberculosis infection (LTBI) remains an under-recognized component of tuberculosis (TB) prevention in South Africa despite its potential to progress to active disease and contribute to ongoing transmission. This study assessed community awareness, perceptions of LTBI treatment, and perceived barriers to screening and care among adults in the King Sabata Dalindyebo Local Municipality, Eastern Cape, South Africa. A community-based cross-sectional survey was conducted using a structured interviewer-administered questionnaire assessing LTBI awareness, perceived risk of progression, understanding of the consequences of untreated LTBI, attitudes towards screening and treatment, and perceived barriers to care. Data were analysed using descriptive statistics, chi-square tests, correlation analysis, and multivariable logistic regression to identify independent predictors of LTBI knowledge. LTBI awareness varied significantly across age groups, with the highest awareness observed among participants aged 20–29 years and lower awareness among older adults. Although concern about progression to active TB was high, knowledge of the consequences of untreated LTBI and uptake of preventive treatment remained limited. Lack of awareness was the most frequently reported barrier to LTBI screening and treatment, followed by fear of medication side effects and stigma. Greater knowledge of LTBI was associated with fewer informational barriers, while lower educational attainment independently predicted poor LTBI knowledge. These findings demonstrate important gaps between awareness, perceived risk, and engagement in preventive care. Strengthening community education, improving access to LTBI information, and addressing informational barriers may enhance screening uptake and preventive treatment in rural, high-burden communities, thereby supporting tuberculosis elimination efforts in South Africa.

## 1. Introduction

Tuberculosis (TB) remains one of the leading infectious causes of morbidity and mortality worldwide. It continues to pose a major public health challenge in South Africa, which ranks among the countries with the highest TB burdens globally [1–3]. Despite substantial progress in TB diagnosis and treatment, transmission remains persistent, particularly in settings characterized by poverty, overcrowding, and a high prevalence of HIV infection. While considerable attention has focused on active TB disease, latent tuberculosis infection (LTBI) represents a substantial but often under-recognized component of the global TB epidemic [4,5]. LTBI is defined as a state in which individuals are infected with *Mycobacterium tuberculosis* but do not exhibit clinical symptoms and are not infectious [4,6]. However, LTBI constitutes a significant reservoir for future active TB disease. Globally, approximately one-quarter of the population is estimated to be infected with *M. tuberculosis*, placing billions of individuals at risk of developing active TB during their lifetime [7]. The lifetime risk of progression from LTBI to active disease is estimated at 5–10% among immunocompetent individuals but is substantially higher among people living with HIV and other immunocompromising conditions [4,8,9]. Consequently, identification and treatment of LTBI have become central components of the World Health Organization (WHO) End TB Strategy and global TB elimination efforts [10]. South Africa continues to experience a substantial burden of LTBI due to its high incidence of TB and HIV co-infection [2,11]. Although precise estimates vary, modeling studies suggest that a considerable proportion of the South African population may harbor latent *M. tuberculosis* infection, particularly among household contacts of TB patients, healthcare workers, and people living with HIV [12,13]. This large reservoir of latent infection contributes to ongoing TB transmission because individuals with untreated LTBI remain at risk of progressing to active disease. In high-burden settings such as South Africa, effective LTBI screening and preventive treatment are increasingly recognized as critical strategies for reducing future TB incidence and achieving national TB control targets [10,14]. Several diagnostic tools are available for identifying LTBI. In South Africa, diagnosis is primarily based on the Tuberculin Skin Test (TST) and Interferon-Gamma Release Assays (IGRAs), particularly among high-risk populations [14,15]. While these tools are available within the healthcare system, implementation of LTBI screening remains challenging in many rural and resource-constrained settings due to financial limitations, laboratory infrastructure requirements, workforce shortages, and competing healthcare priorities [16]. In addition, the successful implementation of LTBI screening programs depends not only on the availability of diagnostic services but also on community awareness, acceptance, and willingness to engage with preventive care. Effective preventive treatment regimens for LTBI are available and have been shown to substantially reduce the risk of progression to active TB disease [10,17]. Early detection through targeted screening and timely initiation of preventive therapy can reduce TB-related morbidity and mortality, interrupt future transmission chains, and contribute to long-term TB elimination efforts [10,18]. However, uptake of LTBI screening and treatment remains suboptimal in many settings. Common barriers include limited public awareness, misconceptions regarding LTBI, stigma, fear of medication side effects, low perceived risk, and challenges accessing healthcare services [19–21]. In South Africa, these barriers may be further exacerbated by socioeconomic inequalities, challenges in healthcare access, and resource constraints within the health system [16,22]. Despite increasing recognition of the importance of LTBI prevention, there remains limited empirical evidence on community-level awareness, perceptions of LTBI treatment, and perceived barriers to screening and preventive care, particularly in rural South African communities. Understanding these factors is essential for developing context-specific interventions that improve uptake of LTBI screening and treatment services and strengthen TB prevention efforts.

Therefore, this study aimed to assess community awareness of latent tuberculosis infection, perceptions of LTBI treatment, and perceived barriers to screening and preventive care among adults residing in the King Sabata Dalindyebo Local Municipality in the Eastern Cape Province of South Africa. By identifying knowledge gaps and barriers to engagement, the study seeks to inform targeted strategies to strengthen LTBI prevention, improve uptake of preventive therapy, and support broader TB control efforts in high-burden rural settings.

## Methods

### Ethics statement

This study was conducted in accordance with the ethical principles of the Declaration of Helsinki and the applicable South African ethical guidelines for research involving human participants. Ethical approval was obtained from the Research Ethics and Biosafety Committee, Faculty of Medicine and Health Sciences, Walter Sisulu University (Reference No. 084/2024). Institutional permission to conduct the study was granted by the Eastern Cape Department of Health (Reference No. EC_202409_008) prior to the commencement of data collection. Written informed consent was obtained from all participants before enrolment. Participants were informed about the purpose of the study, study procedures, potential risks and benefits, and their right to decline participation or withdraw from the study at any stage without penalty or loss of entitled benefits. Participant confidentiality and privacy were strictly maintained by assigning unique study identification numbers and removing all personal identifiers before data analysis. All study data were stored securely and were accessible only to authorised members of the research team. No personally identifiable information was included in any reports, presentations, or publications arising from this study. Participants requiring additional information on tuberculosis prevention, latent tuberculosis infection (LTBI), or available healthcare services were provided with appropriate health education and referral information where necessary.

### Study design and setting

This study employed a cross-sectional, community-based survey design to assess community awareness of latent tuberculosis infection (LTBI), perceptions of LTBI treatment, and perceived barriers to screening and preventive care. The study was conducted in the King Sabata Dalindyebo (KSD) Local Municipality, situated within the Oliver Reginald (O.R.) Tambo District, Eastern Cape Province, South Africa. Data were collected between 10 September and 30 November 2024. The KSD Local Municipality is one of the largest municipalities in the Eastern Cape and includes both peri-urban and predominantly rural communities. The municipality faces substantial socioeconomic challenges, including high levels of unemployment and poverty, limited access to healthcare services, and infrastructure constraints. These factors have been associated with delayed healthcare-seeking behavior, reduced access to preventive health services, and increased vulnerability to infectious diseases, including tuberculosis (TB) and HIV. The O.R. Tambo District continues to experience a high burden of TB and HIV, reflecting broader epidemiological trends observed across the Eastern Cape Province. The coexistence of TB and HIV remains a major public health concern, as HIV infection substantially increases the risk of progression from latent tuberculosis infection to active TB disease. Consequently, effective identification and management of LTBI are critical components of TB prevention efforts within the district. The study setting was purposively selected because of its high TB burden, substantial rural population, and ongoing challenges related to healthcare access and health literacy. These characteristics make the municipality an important setting for understanding community awareness of LTBI and identifying barriers that may influence participation in screening and preventive treatment programmes. Insights gained from this setting may inform the development of context-specific interventions to strengthen LTBI prevention and support broader TB control efforts in rural and underserved communities. A community-based survey approach was considered appropriate because it enabled the assessment of LTBI-related knowledge, perceptions, and barriers among adults residing in the municipality, including individuals who may have limited routine engagement with healthcare services. This approach provided an opportunity to capture community perspectives essential to informing the design and implementation of future LTBI screening, education, and preventive care initiatives.

### Sampling and participants

The study population comprised adult community members residing in the King Sabata Dalindyebo (KSD) Local Municipality, O.R. Tambo District, Eastern Cape Province, South Africa. Eligible participants were adults aged 18 years or older, residents within the study area, and able to provide informed consent. Individuals who declined participation or were unable to communicate effectively with the research team were excluded. Participants were recruited from multiple communities and primary healthcare facilities across the municipality using a community-based sampling approach. Recruitment was conducted through community outreach activities in collaboration with local leaders, community structures, and healthcare facilities. To enhance diversity and reduce selection bias, data collection was conducted at multiple community gathering points, public spaces, and healthcare facilities on different days and times throughout the study period. A probability-based sampling strategy was not feasible because comprehensive community population registers were unavailable, preventing the development of a complete sampling frame. Consequently, an exploratory community-based sampling approach was employed to recruit eligible participants from across the municipality. This approach was considered appropriate given the exploratory nature of the study and the limited availability of local evidence on awareness of latent tuberculosis infection (LTBI), perceptions, and barriers to care in rural South African settings. No formal a priori sample size calculation was performed because insufficient local data were available to inform assumptions regarding expected prevalence estimates, effect sizes, or outcome variability. The final sample consisted of 245 participants and was determined pragmatically based on participant availability, available resources, logistical considerations, and the study timeframe. Although the sample size was considered adequate for descriptive analyses and exploratory assessment of associations, the study may have had limited statistical power to detect smaller effect sizes. Therefore, the findings should be interpreted with caution and viewed as exploratory rather than representative of the wider population. Despite these limitations, the study provides valuable baseline evidence regarding community awareness, perceptions, and barriers related to LTBI in a high TB-burden rural setting and offers an important foundation for future population-based investigations.

### Data collection tool

Data were collected using a structured, interviewer-administered questionnaire designed to assess community awareness, perceptions, and barriers related to latent tuberculosis infection (LTBI). The questionnaire was adapted from the World Health Organization (WHO) tuberculosis education and prevention guidance documents, relevant LTBI literature, and previously published knowledge, attitudes, and practices (KAP) surveys assessing TB-related awareness and health-seeking behaviors [23–25]. The instrument comprised five main domains: (i) socio-demographic characteristics; (ii) awareness and knowledge of LTBI; (iii) perceptions of disease severity and risk; (iv) attitudes towards LTBI screening and preventive treatment; and (v) perceived barriers to LTBI testing, treatment, and healthcare engagement. An overview of the key survey questions and their corresponding domains is presented in Table 1. Questions included both dichotomous (yes/no) and multiple-response items to capture participants’ knowledge, experiences, and perceptions regarding LTBI. Key knowledge questions assessed awareness of LTBI and understanding of the consequences of untreated infection (Q1 and Q6). Perception and attitude questions evaluated concern about progression from LTBI to active tuberculosis, beliefs regarding the necessity of treatment in asymptomatic individuals, and the perceived importance of LTBI screening programmes (Q11–Q13). Additional questions explored perceived barriers to screening and treatment (Q14), previous LTBI screening and treatment experiences (Q15–Q17), and exposure to active TB through close household or community contacts (Q18–Q19) (Table 1). To ensure content validity and contextual relevance, the questionnaire was reviewed by public health researchers and healthcare professionals with expertise in tuberculosis prevention and control. The questionnaire was subsequently translated from English into isiXhosa, the predominant local language in the study area, and independently reviewed by bilingual speakers to ensure conceptual equivalence, cultural appropriateness, and linguistic accuracy. Before data collection, the instrument was pilot tested with a small group of community members who were not included in the final study sample. Feedback from the pilot exercise was used to refine question wording, improve clarity, and enhance respondent understanding. Trained fieldworkers administered the questionnaire through face-to-face interviews to minimize non-response and accommodate participants with varying literacy levels. Responses were recorded electronically and subsequently coded for descriptive, correlation, and multivariable analyses.

**Table 1:** Overview of survey questions assessing awareness, perceptions, screening practices, treatment, and barriers related to latent tuberculosis infection (LTBI).

Question No.	Survey Question	Domain
Q1	Have you heard of latent tuberculosis infection (LTBI) before?	LTBI awareness and knowledge
Q6	What are the possible consequences of untreated LTBI?	LTBI awareness and knowledge
Q11	How concerned are you about progressing from LTBI to active tuberculosis (TB)?	Risk perception
Q12	Do you believe that LTBI treatment is necessary, even if you do not have symptoms?	Treatment attitudes
Q13	How important do you think LTBI screening programmes are?	Screening attitudes
Q14	What barriers may prevent individuals from seeking LTBI testing or treatment?	Perceived barriers
Q15	Have you ever been screened for LTBI?	Screening history
Q16	If you tested positive for LTBI, did you receive treatment?	Treatment history
Q17	If you received LTBI treatment, did you complete the entire course of medication?	Treatment adherence
Q18	Have you ever been in close contact with someone diagnosed with active TB?	TB exposure history
Q19	If yes, did you seek medical evaluation or testing for LTBI?	Health-seeking behaviour

Footnote: LTBI: Latent Tuberculosis Infection; TB: Tuberculosis

### Data analysis

Data were entered, cleaned, and analysed using R version 4.4.1 (R Foundation for Statistical Computing, Vienna, Austria). Descriptive statistics were used to summarize participants’ demographic characteristics, LTBI awareness, perceptions of LTBI treatment, treatment uptake, and perceived barriers to screening and preventive care. Categorical variables were summarized using frequencies and percentages, while continuous variables were described using means and standard deviations (SDs) for normally distributed data or medians and interquartile ranges (IQRs) for non-normally distributed data. The distribution of continuous variables was assessed visually using histograms and quantile–quantile (Q–Q) plots and statistically using the Shapiro–Wilk test. Where assumptions of normality were not met, non-parametric statistical methods were applied. Bivariate analyses were performed to examine associations between demographic characteristics and LTBI-related outcomes. Pearson’s chi-square test was used for categorical variables, while Fisher’s exact test was applied when expected cell counts were less than five. Continuous variables were compared using Student’s t-test for normally distributed variables and the Mann–Whitney U test for non-normally distributed variables. Statistical significance was defined as a two-sided p-value < 0.05. Associations among key LTBI-related constructs, including awareness, concern about progression to active tuberculosis, perceived consequences of untreated LTBI, and perceived barriers to screening and treatment, were assessed using Pearson’s correlation coefficients (r). Corresponding 95% confidence intervals (CIs) and p-values were calculated. Correlation coefficients were subsequently used to generate an exploratory network visualization illustrating patterns of association among study variables. The network analysis was intended to identify relationships among LTBI-related perceptions and barriers and should not be interpreted as evidence of causality. To identify factors independently associated with higher LTBI knowledge and positive attitudes toward LTBI screening and treatment, multivariable logistic regression analysis was performed. Variables demonstrating associations at p < 0.20 in bivariate analyses, together with variables considered epidemiologically relevant based on previous literature, were considered for inclusion in the multivariable model. Adjusted odds ratios (AORs), 95% confidence intervals, and p-values were reported. Model assumptions were assessed before interpreting the results. Multicollinearity among explanatory variables was evaluated using variance inflation factors (VIFs), and model fit was assessed using the Hosmer–Lemeshow goodness-of-fit test. Statistical results are presented as effect estimates, confidence intervals, and p-values to enhance transparency, reproducibility, and interpretability. Detailed results from descriptive, correlation, and multivariable analyses are presented in Tables 2–6–6.

**Table 2:** LTBI awareness by participants’ demographic characteristics.

Variable	LTBI Awareness (%)	95% CI	p-value
Age group			
less than 20 years	40.0	(19.8–64.3)	0.607
20–29 years	52.7	(44.7–60.7)	0.563
30–39 years	44.1	(28.9–60.5)	0.608
40–49 years	25.0	(11.2–46.9)	0.041
50–59 years	15.8	(5.5–37.6)	0.004
greater than or equal to 60 years	18.2	(5.1–47.7)	0.065
Sex			
Male	52.1	(42.1–61.9)	0.757
Female	39.1	(31.7–47.0)	0.009
Ethnicity			
Black African	43.9	(37.8–50.1)	0.063
Coloured	100.0	(20.7–100.0)	1.000

Footnote: LTBI: Latent Tuberculosis Infection; CI: Confidence Interval. LTBI awareness was assessed based on participants’ self-reported knowledge of latent tuberculosis infection. Percentages are presented with corresponding 95% confidence intervals. Results for ethnic subgroups with small sample sizes should be interpreted with caution due to reduced statistical precision.

## Results

### LTBI awareness according to participant demographic characteristics

The results of the descriptive, correlation, and multivariable analyses are presented in Tables 2–6–6. For each analysis, effect estimates, proportions, correlation coefficients, adjusted odds ratios, 95% confidence intervals, and corresponding p-values are reported to facilitate transparent interpretation of the findings. Table 2 presents the distribution of LTBI awareness according to participants’ age, sex, and ethnicity. Overall, awareness of latent tuberculosis infection (LTBI) varied across demographic groups. Awareness was highest among participants aged 20–29 years (52.7%; 95% CI: 44.7–60.7), followed by those aged 30–39 years (44.1%; 95% CI: 28.9–60.5) and those younger than 20 years (40.0%; 95% CI: 19.8–64.3). Lower levels of awareness were observed among older participants, particularly those aged 50–59 years (15.8%; 95% CI: 5.5–37.6) and those aged 60 years and older (18.2%; 95% CI: 5.1–47.7). These findings suggest a declining trend in LTBI awareness with increasing age. Differences in awareness were also observed by sex. Male participants demonstrated higher awareness of LTBI than female participants (52.1%; 95% CI: 42.1–61.9 versus 39.1%; 95% CI: 31.7–47.0). The difference observed among female participants was statistically significant (p = 0.009). Across ethnic groups, awareness was reported by 43.9% of Black participants (95% CI: 37.8–50.1). Although awareness among Coloured participants was estimated at 100.0%, the confidence interval was wide (95% CI: 20.7–100.0), reflecting the small number of participants within this subgroup. Consequently, these estimates should be interpreted with caution. Detailed results are presented in Table 2.

### Perceptions of LTBI treatment, treatment uptake, and perceived barriers to care

Table 3 presents participants’ perceptions of the necessity of LTBI treatment and reported treatment uptake by demographic characteristics. Overall, belief in the importance of LTBI treatment was generally firmer than reported treatment uptake across all demographic groups. Among age groups, participants aged 20–29 years reported the strongest belief in the necessity of LTBI treatment (62.3%; 95% CI: 54.2–69.8; p = 0.004). Similarly, participants younger than 20 years demonstrated relatively high levels of belief in treatment (66.7%; 95% CI: 41.7–84.8), although no uptake of treatment was reported in this subgroup. Participants aged 30–39 years reported moderate belief in treatment (55.9%; 95% CI: 39.5–71.1) and the highest observed treatment uptake (14.8%; 95% CI: 5.9–32.5). Lower levels of belief were observed among participants aged 40–49 years (40.0%; 95% CI: 21.9–61.3) and those aged 60 years and older (45.5%; 95% CI: 21.3–72.0). Despite generally favourable perceptions of LTBI treatment, treatment uptake remained low across all age groups, ranging from 0.0% to 14.8%. These findings suggest that positive attitudes toward LTBI treatment do not necessarily translate into engagement with screening or preventive treatment services. However, these observations should be interpreted with caution because the number of participants reporting a previous LTBI diagnosis and treatment was small. Differences were also observed by sex. Female participants demonstrated slightly higher levels of belief in the necessity of LTBI treatment than male participants (58.9% vs. 58.5%), while treatment uptake remained low in both groups. Similarly, Black participants, who comprised most of the study population, reported moderate-to-high belief in LTBI treatment (58.6%; 95% CI: 52.3–64.6), whereas treatment uptake remained limited (7.1%; 95% CI: 4.2–11.8). Estimates for Coloured participants should be interpreted with caution due to the small sample size and the resulting wide confidence intervals. Participants also reported several barriers that may influence engagement with LTBI screening and treatment services. The most frequently reported barrier was lack of awareness regarding LTBI (62.4%), followed by concerns about medication side effects (14.7%) and stigma associated with tuberculosis (13.9%). Financial constraints were reported less frequently but may nonetheless represent an important obstacle to accessing healthcare services in resource-constrained settings. Overall, the findings suggest that although many participants recognized the importance of LTBI treatment, awareness gaps and perceived barriers may limit participation in screening and preventive treatment programs. Detailed results are presented in Table 3.

**Table 3:** Perceptions of LTBI treatment and reported treatment uptake according to participant demographic characteristics.

Variable	Belief in Treatment (%)	95% CI	p-value	Treatment Uptake (%)	95% CI	p-value
Age group						
less than 20 years	66.7	(41.7–84.8)	0.302	0.0	(0.0–24.2)	0.001
20–29 years	62.3	(54.2–69.8)	0.004	6.2	(3.0–12.2)	<0.001
30–39 years	55.9	(39.5–71.1)	0.608	14.8	(5.9–32.5)	<0.001
40–49 years	40.0	(21.9–61.3)	0.503	6.7	(1.2–29.8)	0.001
50–59 years	57.9	(36.3–76.9)	0.648	8.3	(1.5–35.4)	0.006
greater than or equal to 60 years	45.5	(21.3–72.0)	1.000	0.0	(0.0–43.4)	0.063
Sex						
Male	58.5	(48.4–67.9)	0.121	9.6	(4.7–18.5)	<0.001
Female	58.9	(51.0–66.5)	0.034	5.4	(2.5–11.3)	<0.001
Ethnicity						
Black African	58.6	(52.3–64.6)	0.009	7.1	(4.2–11.8)	<0.001
Coloured	100.0	(20.7–100.0)	1.000	0.0	(0.0–79.3)	1.000

Footnote: LTBI: Latent Tuberculosis Infection; CI: Confidence Interval. Treatment uptake refers to self-reported receipt of LTBI treatment among participants who indicated previous LTBI diagnosis or screening. Confidence intervals for small subgroups should be interpreted with caution due to limited statistical precision.

### Concerns about LTBI, perceived consequences, and barriers to screening and treatment

Table 4 summarizes participants’ concerns regarding progression from latent tuberculosis infection (LTBI) to active tuberculosis, their understanding of the potential consequences of untreated LTBI, and perceived barriers to LTBI screening and treatment. Overall, 75.1% of participants reported being either very concerned or somewhat concerned about the possibility of LTBI progressing to active tuberculosis (95% CI: 69.3–80.1; p < 0.001). This finding suggests a high level of perceived risk associated with LTBI among community members. Despite this concern, only 38.3% of participants correctly identified the full range of consequences associated with untreated LTBI (95% CI: 31.7–45.2; p = 0.001), indicating important knowledge gaps regarding the natural history and public health implications of latent tuberculosis infection. Participants reported several barriers that may influence engagement with LTBI screening and preventive treatment services. The most frequently cited barrier was lack of awareness about LTBI, reported by 62.4% of respondents (95% CI: 56.2–68.3; p < 0.001). This finding is consistent with the relatively low levels of LTBI awareness observed elsewhere in the study and highlights the potential importance of community education and health promotion initiatives. Other barriers identified by participants included concerns about medication side effects, stigma associated with tuberculosis, and financial or healthcare access constraints, although these were reported less frequently. Collectively, these findings suggest that while concern about tuberculosis is common, understanding of LTBI and its consequences remains limited, and lack of awareness may represent an important obstacle to participation in screening and preventive treatment programs.

**Table 4:** Summary of participant concerns, perceived consequences, and barriers related to latent tuberculosis infection.

Indicator	Response	Proportion (%)	95% CI	p-value
Concern about progression to active TB (Q11)	Yes	75.1	(69.3–80.1)	<0.001
Correct identification of consequences of untreated LTBI (Q6)	All the above	38.3	(31.7–45.2)	0.001
Barrier: Lack of awareness (Q14)	Yes	62.4	(56.2–68.3)	<0.001

Footnote: LTBI: Latent Tuberculosis Infection; TB: Tuberculosis; CI: Confidence Interval. Percentages represent the proportion of participants selecting each response category. Confidence intervals were calculated at the 95% level. Statistical significance was assessed using appropriate one-sample proportion tests.

### Correlation analysis of LTBI concerns, perceived consequences, and barriers to screening and treatment

Table 5 presents Pearson correlation coefficients (r), 95% confidence intervals (CIs), and p-values describing the relationships among concerns regarding progression from latent tuberculosis infection (LTBI) to active tuberculosis, perceived consequences of untreated LTBI, and commonly reported barriers to screening and treatment. Most correlations involving concern about LTBI progression were weak and did not reach statistical significance. No significant association was observed between concern about progression and perceived consequences of LTBI (r = −0.089, 95% CI: −0.213 to 0.036; p = 0.167). Similarly, concern about progression was not significantly associated with fear of treatment side effects (r = −0.054, p = 0.397) or stigma (r = 0.040, p = 0.533). A statistically significant positive correlation was observed between perceived consequences of untreated LTBI and lack of awareness (r = 0.186, 95% CI: 0.064–0.307; p = 0.004). However, because the variables were coded as binary indicators, this association should be interpreted cautiously and reflects co-occurrence within the dataset rather than a causal relationship. A significant negative correlation was observed between perceived consequences and fear of side effects (r = −0.201, 95% CI: −0.321 to −0.080; p = 0.002), suggesting that these factors were reported less frequently together. Among perceived barriers, lack of awareness demonstrated moderate inverse correlations with both fear of side effects (r = −0.535, 95% CI: −0.625 to −0.446; p < 0.001) and stigma (r = −0.518, 95% CI: −0.610 to −0.426; p < 0.001). Fear of side effects and stigma were also negatively correlated (r = −0.167, 95% CI: −0.289 to −0.044; p = 0.009). These findings indicate that participants who identified one barrier were less likely to report certain other barriers, suggesting that different obstacles to LTBI screening and treatment may affect different subgroups within the community. Overall, the correlation analysis identified several statistically significant relationships among LTBI-related perceptions and barriers. However, the magnitude of most correlations was weak to moderate, and the findings should be interpreted as exploratory associations rather than evidence of causal pathways.

**Table 5:** Correlation analysis of LTBI concerns, perceived consequences, and barriers to screening and treatment (N = 245).

Variable 1	Variable 2	Correlation Coefficient (r)	95% CI	p-value
Concern about progression	Perceived consequences	-0.089	(-0.213 to 0.036)	0.167
Concern about progression	Lack of awareness	0.119	(-0.005 to 0.243)	0.063
Concern about progression	Fear of side effects	-0.054	(-0.180 to 0.071)	0.397
Concern about progression	Stigma	0.040	(-0.086 to 0.166)	0.533
Perceived consequences	Lack of awareness	0.186	(0.064 to 0.307)	0.004
Perceived consequences	Fear of side effects	-0.201	(-0.321 to -0.080)	0.002
Perceived consequences	Stigma	0.015	(-0.111 to 0.141)	0.813
Lack of awareness	Fear of side effects	-0.535	(-0.625 to -0.446)	<0.001
Lack of awareness	Stigma	-0.518	(-0.610 to -0.426)	<0.001
Fear of side effects	Stigma	-0.167	(-0.289 to -0.044)	0.009

Footnote: LTBI: Latent Tuberculosis Infection; CI: Confidence Interval. Pearson correlation coefficients were calculated using binary-coded variables representing LTBI-related concerns, perceived consequences, and barriers. Correlation coefficients describe the strength and direction of statistical associations and should not be interpreted as evidence of causality. Positive coefficients indicate that variables tended to occur together, whereas negative coefficients indicate inverse relationships.

### Factors associated with LTBI knowledge and attitudes

Table 6 presents the results of the multivariable logistic regression analysis examining factors independently associated with higher LTBI knowledge and more positive attitudes toward LTBI screening and treatment. Variables included in the model were selected based on theoretical relevance and evidence from bivariate analyses. After adjustment for potential confounding factors, participants aged 20–29 years had significantly greater odds of demonstrating higher LTBI knowledge and positive attitudes compared with other age groups (AOR = 1.92, 95% CI: 1.12–3.25; p = 0.018). Female participants were also more likely to report better knowledge and attitudes than male participants (AOR = 1.58, 95% CI: 1.01–2.46; p = 0.043). Higher awareness of LTBI emerged as one of the strongest factors associated with favourable knowledge and attitudes (AOR = 2.14, 95% CI: 1.45–3.95; p < 0.001). Similarly, participants who reported prior contact with an individual diagnosed with tuberculosis had higher odds of demonstrating greater LTBI knowledge and more positive attitudes (AOR = 1.74, 95% CI: 1.02–2.96; p = 0.039). Tertiary education was also independently associated with improved LTBI knowledge and attitudes (AOR = 1.88, 95% CI: 1.15–3.09; p = 0.012). Participants expressing concern about progression from LTBI to active tuberculosis were more likely to demonstrate favourable knowledge and attitudes (AOR = 1.47, 95% CI: 1.03–2.10; p = 0.031). Conversely, identifying lack of awareness as a barrier was independently associated with lower odds of favourable LTBI knowledge and attitudes (AOR = 0.62, 95% CI: 0.41–0.93; p = 0.021). Employment within the healthcare sector was not significantly associated with the outcome after adjustment (AOR = 1.35, 95% CI: 0.82–2.22; p = 0.245). Similarly, fear of treatment side effects (AOR = 0.89, 95% CI: 0.58–1.38; p = 0.604) and stigma-related concerns (AOR = 0.74, 95% CI: 0.49–1.12; p = 0.157) were not significantly associated with LTBI knowledge and attitudes in the adjusted model. The multivariable logistic regression model demonstrated acceptable fit to the observed data (Hosmer–Lemeshow χ² = 6.82, p = 0.338), indicating no evidence of poor model calibration. The model explained approximately 29% of the variability in LTBI knowledge and attitudes (Nagelkerke R² = 0.29) and correctly classified 79.2% of observations. Overall, younger age, female sex, higher LTBI awareness, previous TB contact, tertiary education, and concern about progression to active disease were independently associated with more favourable LTBI knowledge and attitudes. These findings should be interpreted as associations identified within a cross-sectional study and do not imply causal relationships.

**Table 6:** Multivariable logistic regression analysis of factors associated with higher LTBI knowledge and positive attitudes among participants (*N = 245*).

Variable	Adjusted Odds Ratio (AOR)	95% Confidence Interval	p-value
Age 20–29 years	1.92	1.12–3.25	0.018
Female sex	1.58	1.01–2.46	0.043
High LTBI awareness	2.14	1.45–3.95	<0.001
Previous TB contact	1.74	1.02–2.96	0.039
Employment in the healthcare sector	1.35	0.82–2.22	0.245
Tertiary education	1.88	1.15–3.09	0.012
Concern about LTBI progression	1.47	1.03–2.10	0.031
Barrier: Lack of awareness	0.62	0.41–0.93	0.021
Barrier: Fear of side effects	0.89	0.58–1.38	0.604
Barrier: Stigma	0.74	0.49–1.12	0.157

**Model diagnostics:** Nagelkerke R² = 0.29; Hosmer–Lemeshow χ² = 6.82, p = 0.338; Classification accuracy = 79.2%.

Footnote: LTBI: Latent Tuberculosis Infection; TB: Tuberculosis. Adjusted odds ratios were estimated using multivariable logistic regression. Variables were selected based on theoretical relevance and associations observed in bivariate analyses. Odds ratios greater than 1 indicate increased odds of higher LTBI knowledge and positive attitudes, whereas odds ratios less than 1 indicate decreased odds. Given the cross-sectional study design, findings should be interpreted as associations rather than causal effects.

### Correlation network of LTBI concerns, perceived consequences, and barriers to screening and treatment

**Fig 1:**
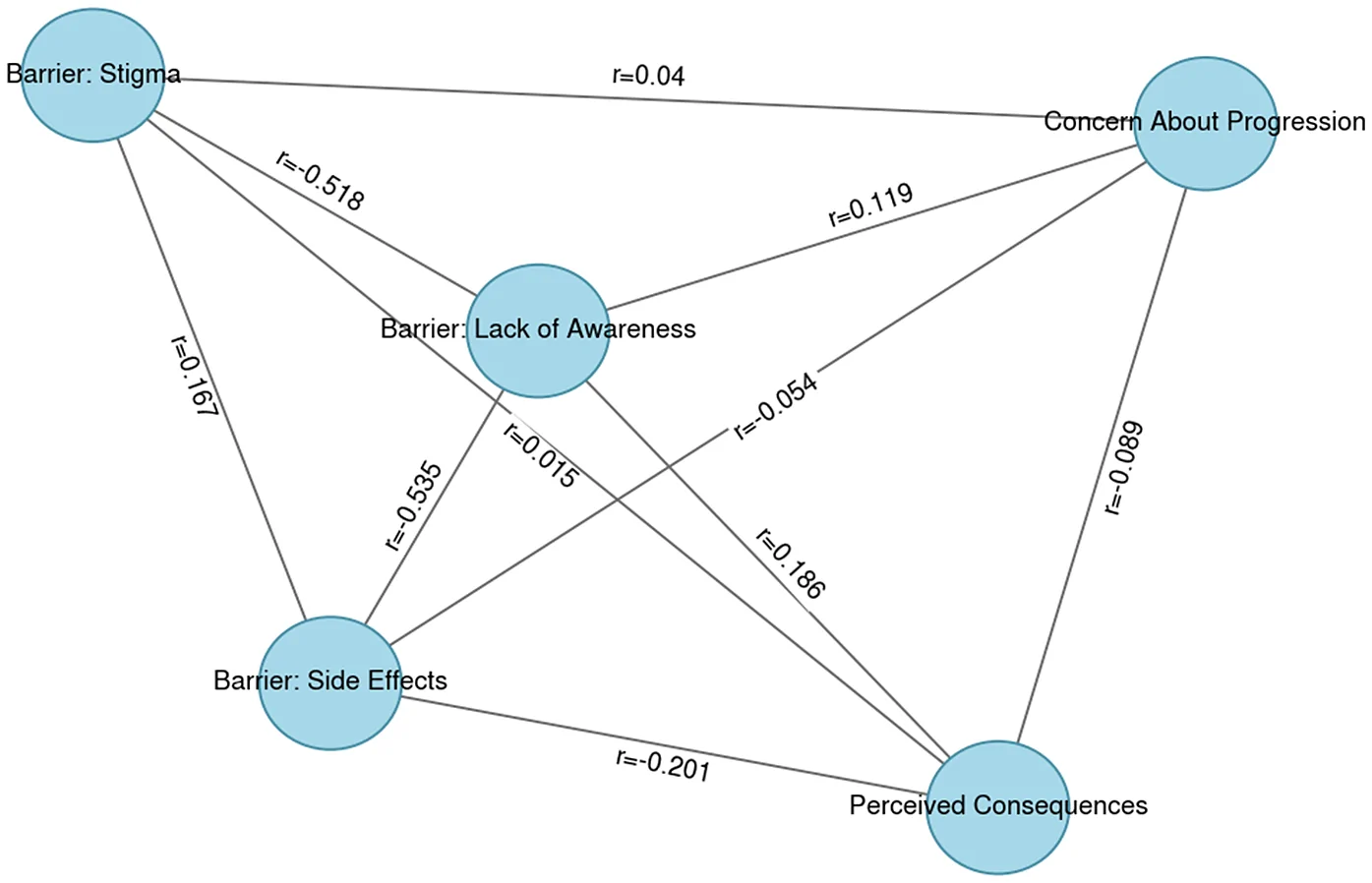
Correlation network of latent tuberculosis infection (LTBI)-related concerns, perceived consequences, and barriers to screening and treatment. Nodes represent key LTBI-related concerns and perceived barriers identified in the study, including stigma, lack of awareness, perceived treatment side effects, concern about progression to active tuberculosis, and perceived consequences of LTBI. Edges represent pairwise correlations between the variables, with the corresponding correlation coefficients (r) displayed along each connection. Positive and negative coefficients indicate the direction of the associations, while the absolute magnitude of r reflects the strength of the relationship. The network illustrates the interrelationships among knowledge, concerns, perceived consequences, and barriers relevant to LTBI screening and treatment. Correlations should not be interpreted as evidence of causal relationships.

Fig 1 presents an exploratory correlation-based network illustrating statistical associations among concern about progression from latent tuberculosis infection (LTBI) to active tuberculosis, perceived consequences of untreated LTBI, and commonly reported barriers to screening and treatment. Consistent with the findings presented in Table 5, most correlations were weak and did not reach statistical significance. No significant association was observed between concern about LTBI progression and perceived consequences of untreated infection (r = −0.089, 95% CI: −0.213 to 0.036; p = 0.167). Similarly, concern about progression was not significantly associated with lack of awareness (r = 0.119, p = 0.063), fear of treatment side effects (r = −0.054, p = 0.397), or stigma (r = 0.040, p = 0.533). A statistically significant positive association was observed between perceived consequences of untreated LTBI and lack of awareness as a reported barrier (r = 0.186, 95% CI: 0.064–0.307; p = 0.004). In addition, significant inverse correlations were identified between lack of awareness and fear of side effects (r = −0.535, p < 0.001) and between lack of awareness and stigma (r = −0.518, p < 0.001). Fear of side effects and stigma were also negatively correlated (r = −0.167, p = 0.009). The network visualization was developed to identify patterns of association among LTBI-related perceptions and barriers and should be interpreted as an exploratory analytical tool. The observed relationships represent statistical correlations rather than causal pathways. Consequently, the network does not imply that one factor leads to another, nor does it represent the direction or flow of information between variables. Rather, it highlights areas where perceptions and barriers co-occurred within the study population and may help identify priorities for future educational and behavioral interventions. Overall, the findings suggest that awareness, understanding of LTBI consequences, and perceived barriers to care are interconnected. However, most associations were weak to moderate in magnitude and should be interpreted cautiously.

**Fig 2:**
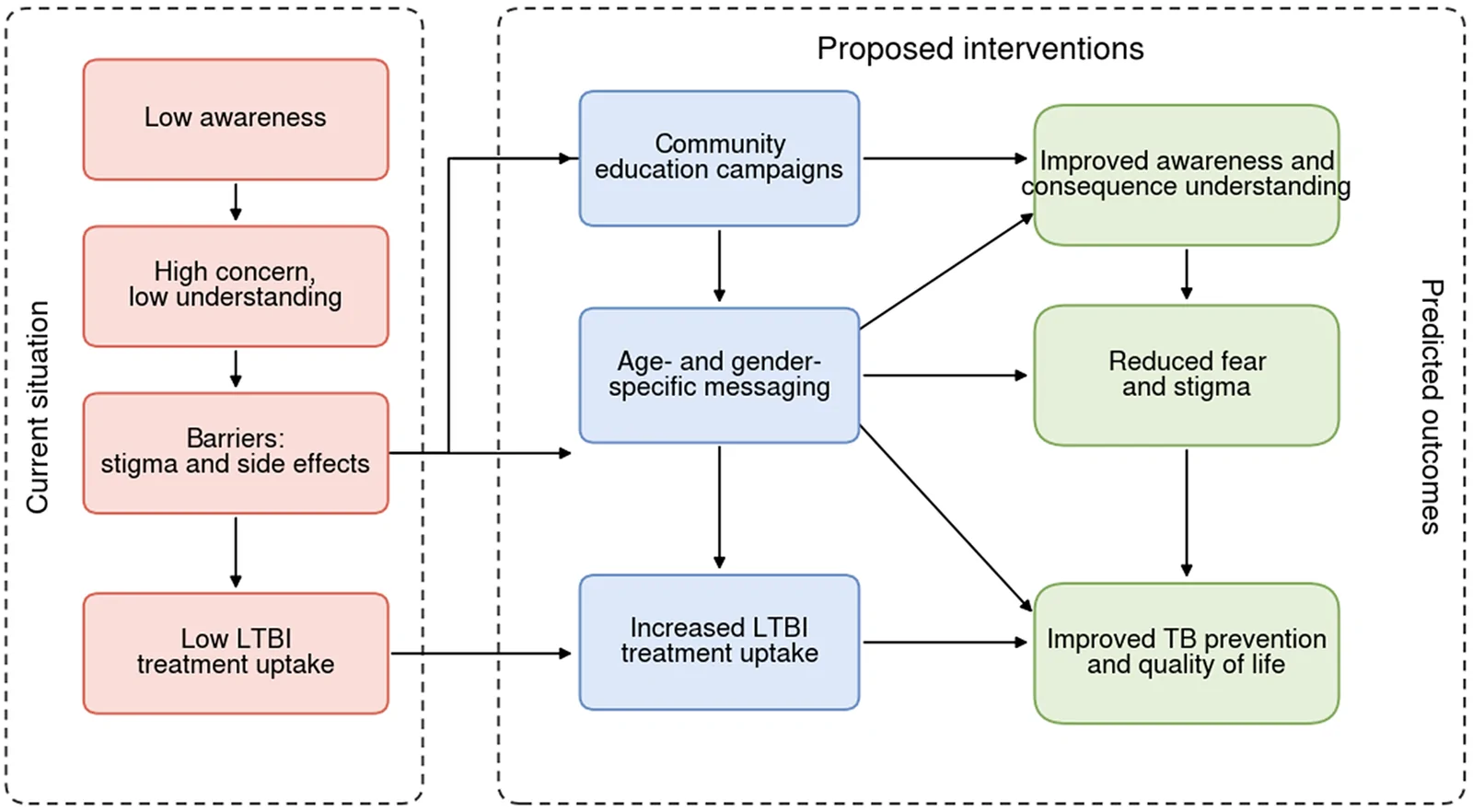
Conceptual framework for potential interventions to improve latent tuberculosis infection (LTBI) awareness, screening, treatment uptake, and prevention in King Sabata Dalindyebo Local Municipality, Eastern Cape, South Africa. The framework integrates key challenges identified in the study with potential intervention strategies and anticipated outcomes. The current situation is characterized by low LTBI awareness, high levels of concern accompanied by limited understanding, perceived barriers including stigma and treatment side effects, and low treatment uptake. Proposed responses include community education campaigns, age- and gender-responsive health messaging, and strategies to strengthen LTBI treatment uptake. These interventions are hypothesized to improve awareness and understanding of LTBI and its consequences, reduce fear and stigma, strengthen engagement with LTBI prevention and treatment, and ultimately contribute to improved tuberculosis prevention and quality of life. Arrows represent proposed conceptual pathways rather than empirically established causal relationships.

The network was constructed using Pearson correlation coefficients derived from participant responses. Nodes represent key LTBI-related constructs, including concern about progression to active tuberculosis, perceived consequences of untreated LTBI, lack of awareness, fear of treatment side effects, and stigma-related barriers. Connecting lines represent the strength and direction of statistical associations between variables. Positive coefficients indicate positive associations, whereas negative coefficients indicate inverse associations. The thickness of the connecting lines corresponds to the magnitude of the correlation coefficient. This network is exploratory in nature and should not be interpreted as a causal framework, temporal sequence, or representation of information flow. Instead, it illustrates patterns of association that may inform future LTBI awareness, education, and screening interventions. The conceptual framework presented in Fig 2 was developed to illustrate how the findings of this study may inform potential intervention priorities to improve awareness of latent tuberculosis infection (LTBI), screening uptake, and engagement in preventive treatment in the King Sabata Dalindyebo (KSD) Local Municipality. The framework is informed by the study findings and existing evidence on barriers to LTBI care and should not be interpreted as a predictive model or a causal pathway. The left side of the framework summarizes key challenges identified in the study, including limited awareness of LTBI, concerns regarding progression to active tuberculosis, and barriers to screening and treatment such as stigma, fear of medication side effects, and healthcare access constraints. Participants commonly reported these factors, which may influence engagement with LTBI prevention services. The central component highlights potential intervention strategies, including community-based education campaigns, community health worker (CHW)-led awareness and stigma-reduction initiatives, and age- and gender-sensitive health promotion approaches. These interventions are proposed as potential responses to the barriers identified in the study. The right side of the framework outlines anticipated public health benefits that may arise if such interventions are successfully implemented, including improved LTBI awareness, reduced perceived barriers to care, increased participation in screening and preventive treatment programs, and strengthened tuberculosis prevention efforts. These proposed outcomes represent potential intervention targets rather than findings directly demonstrated by the present study.

This conceptual framework was developed to illustrate how the findings of the present study may inform potential strategies for improving latent tuberculosis infection (LTBI) awareness, screening uptake, and engagement with preventive treatment in a high TB-burden rural setting. The framework is informed by the study findings, existing evidence on LTBI prevention, and public health principles related to community engagement and health promotion. The framework summarizes key challenges identified among study participants, including limited awareness of LTBI, concerns regarding progression to active tuberculosis, stigma, fear of treatment-related side effects, and barriers to accessing healthcare services. These factors may influence community engagement with LTBI screening and preventive care. The proposed intervention components include community-based health education, community health worker-led awareness and stigma-reduction initiatives, strengthened TB health promotion activities, and age- and gender-sensitive communication strategies. These interventions are presented as potential approaches for addressing the barriers identified in the study. The anticipated outcomes include improved knowledge of LTBI, reduced social and behavioral barriers to screening and treatment, greater participation in preventive care programs, and strengthened tuberculosis prevention efforts. However, these outcomes were not directly evaluated in the present study and are presented as hypothesized benefits based on existing evidence and public health theory. The arrows represent proposed relationships between identified challenges, intervention strategies, and potential outcomes. They do not indicate causality, temporal sequence, or the flow of information and should not be interpreted as empirically validated pathways. Accordingly, the framework should be viewed as a conceptual tool to inform future research, program development, implementation planning, and policy discussions rather than as a predictive or causal model.

## 4. Discussion

The findings of this study have important implications for tuberculosis (TB) prevention in South Africa and other high-burden settings. Although substantial progress has been made in the diagnosis and treatment of active TB disease, prevention remains a critical but often underutilized component of TB control. Latent tuberculosis infection (LTBI) represents a substantial reservoir for future active TB cases, and early identification and preventive treatment are key components of the World Health Organization (WHO) End TB Strategy. Improving community awareness and engagement with LTBI screening and preventive services may therefore help reduce progression to active disease and strengthen TB prevention efforts. This study identified moderate levels of LTBI awareness among community members in the King Sabata Dalindyebo (KSD) Local Municipality, accompanied by substantial concern regarding progression from LTBI to active tuberculosis. However, awareness, concern, and engagement with LTBI-related services were not consistently aligned. Although many participants expressed concern about disease progression and about the consequences of LTBI, understanding of these issues and reported uptake of screening or treatment remained limited. These findings suggest that awareness alone may be insufficient to promote engagement with preventive services and highlight the importance of addressing both knowledge gaps and structural barriers to care. Age-related differences were observed in LTBI perceptions and reported treatment uptake. Participants aged 20–29 years demonstrated the strongest belief in the importance of LTBI treatment, whereas those aged 30–39 years reported the highest treatment uptake. Nevertheless, uptake remained low across all age groups. Similar patterns have been reported in previous studies, suggesting that positive attitudes toward LTBI treatment do not always translate into healthcare-seeking behavior or treatment initiation due to competing social, economic, and health-system factors [13–17]. These findings underscore the importance of distinguishing between favorable perceptions and actual engagement with LTBI prevention services. Lack of awareness emerged as the most frequently reported barrier to LTBI screening and treatment, followed by fear of medication side effects and tuberculosis-related stigma. These findings are consistent with international evidence identifying limited knowledge, stigma, misconceptions, and concerns regarding treatment as important barriers to LTBI care [8,9,15,18,19]. The prominence of awareness-related barriers in this study suggests that educational interventions may play an important role in strengthening community engagement with LTBI prevention programs. The correlation analyses demonstrated several statistically significant associations among LTBI-related perceptions and barriers; however, most correlations were weak to moderate in magnitude. Importantly, concern regarding progression to active tuberculosis was not significantly associated with knowledge of LTBI consequences, fear of treatment side effects, or stigma-related barriers. These findings suggest that emotional concern and factual understanding may represent distinct dimensions of LTBI perceptions within the study population. Because the analyses were cross-sectional and correlation-based, the observed relationships should be interpreted as statistical associations rather than evidence of causal pathways. The positive association observed between perceived consequences of untreated LTBI and awareness-related barriers should also be interpreted cautiously. The correlation analysis does not establish that one factor influences the other. Rather, it highlights patterns of co-occurrence among participant responses and may indicate areas requiring further investigation. Similar findings have been reported elsewhere, where limited health literacy and misconceptions regarding LTBI have been associated with reduced engagement in preventive services [20–23].

The conceptual framework presented in Fig 2 was developed to synthesize the study findings and identify potential intervention priorities. Unlike the correlation network, which was derived directly from the study data, the conceptual framework is not an empirical model and should not be interpreted as a validated causal pathway. Instead, it provides a programmatic illustration of how identified barriers, including limited awareness, stigma, concerns regarding treatment, and healthcare access challenges, may be addressed through community-based interventions. Proposed strategies include health education campaigns, community health worker (CHW)-led awareness initiatives, stigma-reduction activities, and age- and gender-sensitive communication approaches. These interventions are supported by existing evidence and may be relevant for strengthening LTBI prevention efforts in rural settings [24–27]. The findings should also be interpreted within the broader South African context. South Africa continues to experience one of the highest burdens of TB and HIV globally, and LTBI remains an important but often under-recognized component of the epidemic. Diagnostic tools for LTBI, including the Tuberculin Skin Test (TST) and Interferon-Gamma Release Assays (IGRAs), are available within the healthcare system, particularly for high-risk populations. However, implementation of LTBI screening remains uneven, especially in rural and resource-constrained settings, where limited healthcare access, infrastructure constraints, workforce shortages, and competing priorities may restrict uptake. In districts such as O.R. Tambo, strengthening community awareness and acceptance of LTBI screening and preventive treatment may contribute to earlier identification of individuals at risk of developing active TB and support broader TB prevention goals. Overall, the findings highlight the importance of integrating LTBI education, screening, and preventive services into routine primary healthcare and community-based TB programs. Future interventions should address both informational and structural barriers to care and consider demographic differences in perceptions and engagement with LTBI prevention strategies.

### Limitations

Several limitations should be considered when interpreting the findings of this study. First, the cross-sectional study design precludes the assessment of temporal relationships and prevents causal inference. Consequently, the observed relationships between LTBI awareness, perceptions, attitudes, and barriers should be interpreted as associations rather than cause-and-effect relationships. Second, this study was exploratory in nature and employed a community-based sampling approach within a single municipality. No formal a priori sample size or statistical power calculation was conducted because limited local data were available to inform the assumptions required for such calculations. The final sample of 245 participants was determined pragmatically based on feasibility, available resources, and the study timeframe. Although the sample size was sufficient for descriptive and exploratory analyses, the study may have been underpowered to detect smaller associations, and some estimates may therefore be less precise. Third, the study relied on self-reported information, which may be affected by recall bias, social desirability bias, and potential misclassification of participants’ knowledge, attitudes, and experiences related to LTBI. Fourth, because the study was conducted in a predominantly rural setting within the King Sabata Dalindyebo Local Municipality, the findings may not be generalizable to urban populations or other regions of South Africa with different demographic, socioeconomic, and healthcare contexts. Finally, the conceptual framework presented in Fig 2 was developed to synthesize the study findings and existing literature and was not empirically tested within the present study. The proposed intervention pathways should therefore be regarded as hypothesis-generating and intended to inform future research, programme development, and policy discussions rather than as validated causal mechanisms. Despite these limitations, this study provides important baseline evidence regarding community awareness, perceptions, and barriers related to LTBI in a rural South African setting. The findings contribute to the limited body of evidence on LTBI in high TB-burden communities and may help inform future interventions aimed at strengthening LTBI awareness, screening, and preventive treatment uptake.

### Recommendations

Based on the findings of this study, several recommendations may be considered to strengthen LTBI awareness, screening uptake, and engagement with preventive treatment in rural and high TB-burden settings.

### Strengthen community-based LTBI education

Community-based health education programs should be developed and implemented to improve public understanding of LTBI, its relationship to active tuberculosis, and the benefits of preventive treatment. Educational materials should be culturally appropriate, linguistically accessible, and tailored to local contexts. Particular emphasis should be placed on addressing misconceptions regarding LTBI, explaining the risk of progression to active disease, and promoting the importance of preventive care among asymptomatic individuals.

### Enhance community health worker engagement and stigma reduction

Community health workers (CHWs) may play an important role in improving LTBI awareness and facilitating engagement with screening and preventive treatment services. Integrating LTBI education into existing community TB programs and stigma-reduction initiatives may help address misconceptions, increase trust in healthcare services, and support informed decision-making. Efforts to reduce TB-related stigma should be incorporated into broader community health promotion activities.

### Strengthen integration of LTBI services within existing TB programs

LTBI education, screening, and preventive treatment should be further integrated into existing tuberculosis and primary healthcare services, particularly for populations at increased risk of progression to active disease, including household contacts of TB patients and people living with HIV. Improving accessibility of LTBI services may help support earlier identification and management of latent infection.

### Address health system and access barriers

Health system readiness for expanded LTBI screening and preventive treatment should be assessed, particularly in rural and resource-constrained settings. Consideration should be given to workforce capacity, training requirements, continuity of care, referral systems, and patient follow-up mechanisms. Strategies to address logistical barriers, including transportation challenges and healthcare accessibility, may further support engagement with LTBI services.

### Support future research and innovation

Further research is needed to understand better behavioral, social, and health-system factors influencing LTBI screening and treatment uptake in South Africa. Future studies should include larger and more diverse populations, evaluate the effectiveness of community-based interventions, and explore the potential role of digital health technologies in supporting LTBI education, follow-up, and treatment adherence. Longitudinal studies would be particularly valuable for examining how awareness and perceptions influence engagement with LTBI prevention services over time.

## Conclusion

This study provides important insights into community awareness, perceptions, and barriers related to latent tuberculosis infection in a rural South African setting. Although many participants expressed concern regarding progression from LTBI to active tuberculosis, awareness, understanding of LTBI consequences, and engagement with screening and preventive treatment services remained variable. Lack of awareness, concerns regarding treatment, and stigma emerged as important barriers to LTBI care. The findings suggest that improving LTBI awareness alone may be insufficient to increase engagement with preventive services. Instead, comprehensive approaches that combine community education, stigma reduction, community health worker involvement, and improved access to healthcare services may be required to strengthen LTBI prevention efforts. Given the cross-sectional nature of the study, the observed relationships should be interpreted as associations rather than causal effects. Nevertheless, the findings highlight opportunities to strengthen LTBI education and preventive services within existing tuberculosis control programs. Addressing identified knowledge gaps and barriers may increase uptake of screening and preventive treatment and support broader efforts to reduce the burden of tuberculosis in South Africa and other high-burden settings.
