# Supplementary material for: Latent tuberculosis infection in focus: Evaluating public concerns, treatment perceptions, and awareness in King Sabatha Dalindyebo Local Municipality, Eastern Cape, South Africa
Source: PLOS Glob Public Health. 2026 Sep 15;6(9):e0005130. doi: 10.1371/journal.pgph.0005130 (PMC13577523; doi:10.1371/journal.pgph.0005130)
Supplement: Supplementary file 1 [file pgph.0005130.s001.docx]

Latent Tuberculosis Infection in Focus: Evaluating Public Concerns, Treatment

Perceptions, and Awareness in King Sabatha Dalindyebo Local Municipality, Eastern

Cape, South Africa

1. Does this manuscript meet publication criteria? Is the manuscript technically sound, and do the data support the conclusions? The manuscript must describe methodologically and ethically rigorous research with conclusions that are appropriately drawn based on the data presented.

Yes, it meets the criteria. But it can be improved. Other statistical model analysis can be explored to check for factors to identify variables affecting the knowledge and attitude, controlling for the confounders.

2. Has the statistical analysis been performed appropriately and rigorously?

Yes, but can be improved as mentioned above.

3. Have the authors made all data underlying the findings in their manuscript fully available (please refer to the Data Availability Statement at the start of the manuscript PDF file)?

The statement is given. However, tables are missing with the manuscript submission.

The PLOS Data policy requires authors to make all data underlying the findings described in their manuscript fully available without restriction, with rare exception. The data should be provided as part of the manuscript or its supporting information, or deposited to a public repository. For example, in addition to summary statistics, the data points behind means, medians and variance measures should be available. If there are restrictions on publicly sharing data—e.g. participant privacy or use of data from a third party—those must be specified.

4. Is the manuscript presented in an intelligible fashion and written in standard English?

Yes, some sentences are ambiguous and can be improved

The journal does not copyedit accepted manuscripts, so the language in submitted articles must be clear, correct, and unambiguous. Any typographical or grammatical errors should be corrected at revision, so please note any specific errors here.

5. PLOS authors have the option to publish the peer review history of their article. If published, this will include your full peer review and any attached files.

Do you want your identity to be public for this peer review? If you choose “no”, your identity will remain anonymous but your review may still be made public.

No

For information about this choice, including consent withdrawal, please see our [Privacy Policy.](https://https/plos.org/privacy-policy/)

7. Do you have any potential or perceived competing interests that may influence your review? Please review our Competing Interests policy and declare any potential interests. If you have no competing interests, please write "I have no competing interests."

I have no competing interests

8. Did you receive any assistance in preparing this review (e.g. from a post-doc or graduate student)? If yes, please include their name below. (optional)

No

9. If accepted, do you think this submission should be highlighted on the journal website and/or to the media? (optional)

No

10. Recommendation term (Accept / Minor Revision / Major Revision / Reject / Proceed without Recommendation)

Major Revision. And please re-submit with all the required changes and with the tables for review.

11. Do you want your identity to be public for this peer review? (Yes / No)

No
