# Supplementary material for: Latent tuberculosis infection in focus: Evaluating public concerns, treatment perceptions, and awareness in King Sabatha Dalindyebo Local Municipality, Eastern Cape, South Africa
Source: PLOS Glob Public Health. 2026 Sep 15;6(9):e0005130. doi: 10.1371/journal.pgph.0005130 (PMC13577523; doi:10.1371/journal.pgph.0005130)
Supplement: Supplementary file 2 [file pgph.0005130.s002.docx]

**Latent Tuberculosis Infection in Focus: Evaluating Public Concerns, Treatment**

**Perceptions, and Awareness in King Sabatha Dalindyebo Local Municipality, Eastern**

**Cape, South Africa**

Lindiwe Modest Faye, Ncomeka Sineke, Ntandazo Dlatu, Urgent Tsuro, Mojisola Clara

Hosu and Teke Apalata

Corresponding author: Lindiwe Modest Faye (lfaye@wsu.ac.za)

**Abstract**

Background: Latent tuberculosis infection (LTBI) remains an overlooked aspect of

tuberculosis (TB) control in South Africa, despite its potential to develop into active

disease. This study aimed to assess public awareness, perceptions of treatment, and

perceived barriers to LTBI care in a high-burden rural setting.

Methods: A cross-sectional, community-based survey was conducted among adults in the

King Sabatha Dalindyebo Local Municipality, Eastern Cape. A structured questionnaire

assessed knowledge of LTBI, concerns about progression, perceived consequences, and

treatment-related barriers. Data were analyzed using descriptive statistics, chi-square tests,

and Pearson correlation, with results presented by demographic subgroup.

Results: Awareness of LTBI varied significantly by age, with the highest awareness among

20–29-year-olds (52.7%; 95% CI: 44.7–60.7; p = 0.5625) and the lowest in those aged 50–

59 (15.8%; 95% CI: 5.5–37.6; p = 0.0044). Belief in the necessity of treatment was

strongest in the <20 group (66.7%) and those aged 20–29 (62.3%; 95% CI: 54.2–69.8; p =

0.0036), though actual treatment uptake remained low across all age groups, peaking at

14.8% (95% CI: 5.9–32.5; p = 0.0003) in the 30–39-year-old cohort.

Concern about LTBI progressing to active TB was widespread (75.1%; 95% CI: 69.3–80.1;

p < 0.0001), but only 38.3% correctly identified the full consequences of untreated LTBI

(p = 0.0012). The most commonly cited barrier was lack of awareness (62.4%; 95% CI:

56.2–68.3; p < 0.0001). Correlation analysis revealed a weak but significant association

between understanding of consequences and reduced informational barriers (r = 0.186;

95% CI: 0.064–0.307; p = 0.0035), while emotional concern was not significantly

associated with actual knowledge or behavior.

Conclusion: The findings highlight a disconnect between concern and action in LTBI care.

While awareness and worry are common, they are insufficiently grounded in accurate

knowledge or translated into treatment uptake. Community education, stigma reduction,

and age-sensitive messaging are crucial for enhancing LTBI engagement and supporting

TB prevention in rural South Africa.

Keywords: Latent tuberculosis infection, awareness, treatment perception, stigma, rural

health, barriers, TB prevention

**Introduction**

Tuberculosis (TB) remains a critical public health challenge in South Africa, a country that

continues to bear one of the highest burdens of TB globally [1, 2, 3]. While much attention

is paid to active TB, latent tuberculosis infection (LTBI) represents a silent yet significant

component of the disease burden. LTBI refers to a state in which individuals are infected

with Mycobacterium tuberculosis but do not exhibit clinical symptoms and are not

infectious [4,5]. However, approximately 5–10% of individuals with LTBI progress to

active TB in their lifetime, with this risk being markedly higher among those with

immunosuppression, including HIV infection [4,6,7]. Despite the availability of diagnostic

and preventive treatment strategies, LTBI remains poorly addressed in many high-burden,

resource-limited settings. Challenges include limited awareness among the public and

healthcare providers, stigma, fear of side effects, and barriers to accessing treatment

[8,9,10]. In South Africa, these challenges are compounded by social inequalities,

inadequate health infrastructure, and limited community engagement around preventive

TB strategies [11,12].

This study focuses on assessing community-level concerns, awareness, and perceptions of

treatment related to LTBI in the King Sabatha Dalindyebo (KSD) Local Municipality,

Eastern Cape. By evaluating public understanding and identifying barriers to engagement

with LTBI care, this research aims to inform context-specific interventions to improve

LTBI diagnosis, treatment uptake, and TB prevention outcomes.

**Methodology**

Study design and setting

This was a cross-sectional, community-based survey conducted in the KSD Local

Municipality, situated in the Oliver Reginald (O.R.) Tambo District of the Eastern Cape

Province. The area is characterized by a high burden of TB and HIV, limited healthcare

access in rural settlements, and persistent social inequalities affecting health-seeking

behavior. The study was conducted between 10 September and November 2024.

Sampling and participants

Participants were recruited using random sampling from various communities in the

municipality and clinics across the municipality. The inclusion criteria were individuals

aged 18 years and above, residing in the KSD Local Municipality, and able to provide

informed consent.

Data collection tool

Table 1 outlines the structured questionnaire items administered to participants in the KSD

Local Municipality. Questions assessed awareness of LTBI, perceived consequences,

emotional concern, treatment beliefs, testing history, and barriers to care. The tool was

adapted from World Health Organization (WHO) TB education guidelines, the tool

covered key domains including demographic characteristics, awareness and understanding

of LTBI (e.g., Q1, Q6), attitudes toward LTBI treatment (Q11–Q13), perceived barriers to

testing and treatment (Q14), and history of LTBI screening or treatment (Q15–Q19) (Table

1). To ensure cultural relevance and clarity, the questionnaire was translated into isiXhosa

for linguistic and contextual appropriateness.

Data analysis

Descriptive statistics were used to summarize demographic variables and LTBI-related

knowledge and attitudes. Proportions, 95% confidence intervals (CIs), and p-values were

calculated to assess significant differences across age, gender, and ethnicity subgroups.

Binary variables were generated for multivariate analysis and correlation testing. Pearson’s

correlation coefficients (r), 95% CIs, and p-values were computed to evaluate associations

between concern, awareness, and perceived barriers. A network visualization was

constructed using correlation weights to illustrate the strength of relationships among key

constructs.

Ethical considerations

The study was conducted following the Declaration of Helsinki and approved by the

Research Ethics and Biosafety Committee of the Faculty of Medicine and Health

Sciences of Walter Sisulu University (ref. no. 084/2024) and the Eastern Cape

Department of Health (ref. No. EC_202409_008).

Results

Table 2 (supp file 2) presents an analysis of awareness and treatment perceptions related

to LTBI across demographic groups, including age, gender, and ethnicity. Awareness of

LTBI varied notably across age groups. Out of 245 participants, the highest awareness was

observed in participants aged 20–29 years (52.7%; 95% CI: 44.7–60.7), whereas

individuals aged 60 and above had the lowest awareness (18.2%; 95% CI: 5.1–47.7).

Although younger participants (<20 years) had moderate awareness (40.0%), their belief

in treatment necessity and uptake was minimal. Gender and ethnicity trends followed

similar patterns, though statistically significant differences were mainly seen in age-based

comparisons.

In Table 3 (supp file 2), participants aged 20–29 years demonstrated the highest belief in

the necessity of LTBI treatment (62.3%; 95% CI: 54.2–69.8; p = 0.0036), However, actual

treatment uptake in this group remained low (6.2%), reflecting a disconnect between

awareness and action. The 30–39-year group exhibited moderate belief (55.9%) but

reported the highest treatment uptake (14.8%; 95% CI: 5.9–32.5). In contrast, the 40–49

and 60+ age groups reported relatively low belief in treatment (40.0% and 45.5%,

respectively), with minimal or no uptake, particularly among those aged 60 and above,

where uptake was 0%. Among participants under 20 years, belief in treatment was

relatively high (66.7%), but no uptake was recorded. Gender-specific patterns, though not

fully tabulated here, indicated that females generally reported stronger belief in treatment

necessity and slightly higher treatment uptake. Ethnicity trends, primarily among Black

South African respondents, the dominant demographic in this sample, mirrored broader

findings of moderate to high treatment perception but consistently low uptake. Notably,

the most frequently cited barrier to LTBI testing or treatment was a lack of awareness

(62.4%), followed by fear of side effects (14.7%) and stigma associated with TB (13.9%).

Financial constraints were reported less often but remain a potential obstacle.

Table 4 (supp file 2) summarizes key findings on public concerns, perceived consequences,

and barriers to LTBI treatment. Approximately 75.1% of participants reported being either

very or somewhat concerned about the risk of LTBI progressing to active tuberculosis

(95% CI: 69.3–80.1; p < 0.0001. However, only 38.3% accurately identified the full range

of consequences associated with untreated LTBI (95% CI: 31.7–45.2). The most prevalent

barrier to testing and treatment was lack of awareness, cited by 62.4% of respondents (95%

CI: 56.2–68.3; p < 0.0001).

Table 5 (supp file 2) presents Pearson correlation coefficients (r), confidence intervals (CI),

and p-values for pairwise associations among community concerns about LTBI

progression, understanding of its health consequences, and three commonly cited barriers

to testing or treatment (lack of awareness, fear of side effects, and stigma). Statistically

significant associations are highlighted, with the strongest positive correlation observed

between perceived consequences and lack of awareness (r = 0.186, p = 0.0035), suggesting

that improved understanding may reduce informational barriers.

Fig.1 (Supp file 1) is a correlation analysis conducted to explore the relationships among

concern about LTBI progression, perceived consequences of untreated infection, and the

three most frequently cited barriers to testing or treatment. The only statistically significant

correlation was observed between perceived consequences and lack of awareness as a

barrier (r = 0.186; 95% CI: 0.064–0.307; p = 0.0035). No statistically significant

associations were found between concern about progression and any of the other variables,

including perceived consequences (r = -0.089; p = 0.1668), lack of awareness (r = 0.119;

p = 0.0634), fear of side effects (r = -0.054; p = 0.3974), or stigma (r = 0.040; p = 0.5331).

The Predictive pathway of LTBI Intervention Impact in Figure 2 (Supp file 1) illustrates

the theorized pathway from the current community-level challenges in the KSD Local

Municipality to potential improvements in LTBI care and TB prevention. On the left, the

model highlights key obstacles to LTBI treatment uptake, including low awareness, high

concern with limited understanding, and persistent barriers such as stigma, fear of side

effects, and restricted access to healthcare. These factors collectively contribute to poor

engagement with LTBI screening and preventive therapy. At the center, three targeted

interventions community education campaigns, stigma reduction through community

health worker (CHW) involvement, and age- and gender-sensitive health messaging are

proposed to directly address these root causes. The right side of the model projects the

anticipated outcomes if such interventions are implemented: enhanced community

knowledge of LTBI, reduced social and psychological barriers, increased uptake of

treatment, and ultimately, improved TB prevention and quality of life in the affected

population.

**Discussion**

This study highlights a complex and often fragmented understanding of LTBI among

community members in the KSD Local Municipality. While general awareness of LTBI

was moderate, and concern about disease progression was high, these did not consistently

translate into an accurate understanding of consequences or meaningful engagement with

testing and treatment. Participants aged 20–29 exhibited the highest belief in the necessity

of LTBI treatment, while those aged 30–39 had the highest actual uptake, although both

rates remained low [13,14]. Various studies on LTBI demonstrated different patterns of

belief in the need for treatment and how often people start it across various age groups. For

example, while younger adults may see LTBI treatment as more necessary, actual

participation can be affected by a complex mix of factors, with other age groups possibly

showing higher completion rates because of different motivations or fewer obstacles

[15,16,17]. These findings suggest a gap between intention and behavior regarding LTBI

treatment, likely driven by a combination of knowledge gaps, social stigma, and systemic

barriers [18,16,9]. This is further supported by the strong identification of lack of

awareness (62.4%) as a leading barrier, followed by fear of side effects (14.7%) and TB related stigma (13.9%). Such specific findings align with broader literature emphasizing

these as significant impediments to LTBI care [19,15]. Correlation and network analyses

reinforced this disjunction. While concern about progression was high (75.1%), it was not

significantly associated with accurate knowledge or barrier-related perceptions [20]. The

only statistically significant correlation observed was between perceived consequences and

lack of awareness (r = 0.186, p = 0.0035), suggesting that improved understanding may

reduce informational barriers [17, 21]. The study by Magwaza et al. also highlights

similarities in the role of "ever heard of LTBI before" and its negative correlation with

understanding progression, which is consistent with our findings regarding "lack of

awareness" and "perceived consequences [17]." These findings underscore the need for

integrated community education campaigns that move beyond raising general awareness

to addressing the specific misconceptions and fears associated with LTBI. Tailored

messaging, stigma reduction interventions, and community health worker engagement may

be necessary to close the gap between perception and action. Furthermore, interventions

should be stratified by age and gender to ensure context-specific relevance, with particular

focus on empowering younger adults and supporting older, at-risk individuals.

The correlation network visually represents the relationships among community concern

about LTBI progression, understanding of its health consequences, and perceived barriers

to testing or treatment. Nodes represent key constructs, concerns, perceived consequences,

and three prominent barriers, while edges indicate the strength and direction of their

statistical association (based on Pearson’s correlation coefficients).

The most notable connection in the network is between Perceived Consequences and

Barrier is Lack of Awareness (r = 0.186), the only statistically significant correlation (p =

0.0035) [17]. This edge suggests that individuals who have a better understanding of the

consequences of untreated LTBI are also more likely to be aware of LTBI itself,

highlighting the foundational role of comprehensive health knowledge in overcoming

informational barriers. These findings are consistent with similar results in Canada, among

Chinese immigrants, and in three Southeastern counties in the United States [22,23].

Conversely, the central node's concern about progression is only weakly connected to other

variables in the network. Its low or nonsignificant correlations with perceived

consequences, fear of side effects, and stigma (all p > 0.05) suggest that emotional concern

about disease progression may exist independently of factual understanding or perceived

structural barriers [24]. This separation implies that concern may be driven by generalized

fear or hearsay rather than informed risk assessment. Additionally, the lack of significant

association between concern and stigma contradicts assumptions that fear of TB-related

discrimination directly influences anxiety about disease progression. Similarly, the weak

connection between concern and side effects indicates that worry about progression does

not strongly predict fear of treatment harms [25,26]. Besides perceptions and uptake, this

study evaluated public concern regarding LTBI progression, understanding of its

consequences, and barriers to testing or treatment. A significant proportion of respondents

expressed themselves as being either very concerned or somewhat concerned about

progression from LTBI to active TB. This indicates a general awareness of TB risks. When

asked about the consequences of untreated LTBI, most participants correctly identified

serious health implications, including disease progression and transmission. However, this

concern and understanding did not necessarily translate into treatment uptake.

The predictive model reinforces findings from this study, highlighting a significant

disconnect between concern and action regarding LTBI care. While participants express

anxiety about disease progression, this concern is not grounded in factual understanding or

accompanied by proactive behavior, such as testing or treatment uptake. This disconnect

between perceived risk and preventive behavior, such as testing, treatment initiation, or

adherence, is both revealing and consistent with findings from other studies. A similar

behavioral disconnect was observed by Alsdurf et al., who described a cascade of care

model for LTBI and noted significant attrition at each stage from testing to treatment

completion. They attributed this to low health literacy, poor risk perception, and systemic

barriers, particularly in vulnerable populations. Despite the worry about progression to

active TB, patients failed to act, often due to inadequate understanding of LTBI and its

management [27]. In a South African context, a study by Louw reported that community

members who feared TB infection often delayed testing and treatment because their

concerns were not rooted in accurate knowledge [28]. Their study underscored that

misconceptions and low perceived personal risk contributed to reduced uptake of

preventive therapy, even among household contacts of active TB patients. Contrastingly, a

study conducted in Ethiopia found that TB-related knowledge strongly predicted testing

behavior. In communities with targeted education campaigns, individuals were more likely

to take preventive action when they understood LTBI transmission and treatment

implications. This suggests that contextual and educational interventions can realign

concern with evidence-based action. Moreover, Zenner et al. emphasized in their UK-based

LTBI screening program that psychological readiness and informed consent were crucial

for high uptake. Participants who received tailored health information were more willing

to participate in testing and complete treatment. Together, these studies illustrate that while

emotional concern may exist, actionable engagement hinges on an accurate understanding

and access to supportive services. This reinforces the need for interventions that do more

than raise alarm but must empower individuals with practical knowledge and resources

[29].

The model suggests that community-level education is essential to convert emotional

concern into informed decision-making. This behavioral inertia can largely be attributed to

gaps in health literacy, especially in resource-limited and rural settings. Here, community level education becomes an essential lever for change. Community-based education can close knowledge gaps by explaining LTBI risk, treatment options, and outcomes [30]; Address

stigma and misconceptions, especially in rural settings where TB is often misunderstood;

Promote agency, empowering individuals to act on their concerns and enhance healthcare

trust, particularly when delivered by community health workers. In Ethiopia, Datiko et al,

found that empowering health extension workers to engage directly with community

members increased awareness and testing uptake [31]. Additionally, integrating

community health workers and reducing LTBI- and TB-related stigma are likely to enhance

trust and access, particularly for women, youth, and older adults who face different sets of

challenges [32].

Tailored communication is especially relevant, as the study revealed age-specific patterns:

younger respondents were more concerned but less likely to act, while those aged 30–39

had higher follow-through rates. This suggests that uniform strategies may be ineffective

without considering demographic context [33]. The projected endpoint, which improves

quality of life and TB prevention, aligns with South Africa’s national TB control priorities

and the WHO End TB Strategy. The model provides a foundation for operationalizing

study insights into practical, scalable interventions that can bridge the gap between

awareness and preventive action in high-burden, rural settings. In summary, the network

reveals that while concern is widespread, it is not well integrated with accurate knowledge

or perceptions of barriers. This disjunction underscores the need for interventions that

simultaneously improve LTBI literacy, reduce informational barriers, and ensure concerns

are grounded in accurate health understanding.

**Recommendations**

Based on the findings of this study, several targeted strategies are recommended to

strengthen LTBI care in rural, high-burden settings such as the KSD Local Municipality.

First, community-based education campaigns should be prioritized to improve awareness

of LTBI, its potential progression, and the importance of treatment even in asymptomatic

individuals. These messages must be tailored by age and gender to address the differing

perceptions and uptake behaviors identified across demographic groups. Second, stigma

reduction initiatives should be integrated into TB outreach, using trusted CHWs to build

local trust and dispel misconceptions around LTBI testing and treatment. Third, policylevel

support is needed to ensure the inclusion of LTBI education in existing TB prevention

programs and to remove logistical barriers such as limited-service availability or

transportation challenges. Lastly, further research should explore health system readiness

to deliver LTBI preventive therapy at scale and investigate the role of digital platforms in

reinforcing community engagement.

**Conclusions**

This study highlights a significant gap between public concern and action regarding LTBI

in a rural South African setting. While many community members worry about LTBI

developing into active TB, this concern is not backed by sufficient knowledge, and it does

not lead to high treatment rates. Low awareness, misconceptions about the consequences,

and persistent social barriers such as stigma and fear of side effects continue to hinder

engagement with LTBI services. Correlation analysis shows that accurate knowledge is a

better predictor of reducing barriers than concern alone, emphasizing the importance of

informed health literacy as a catalyst for behavior change. Addressing these gaps through

culturally appropriate, community-led interventions will be crucial for improving TB

prevention efforts and health outcomes among vulnerable populations.
