# Supplementary material for: Latent tuberculosis infection in focus: Evaluating public concerns, treatment perceptions, and awareness in King Sabatha Dalindyebo Local Municipality, Eastern Cape, South Africa
Source: PLOS Glob Public Health. 2026 Sep 15;6(9):e0005130. doi: 10.1371/journal.pgph.0005130 (PMC13577523; doi:10.1371/journal.pgph.0005130)
Supplement: Supplementary file 3 [file pgph.0005130.s003.docx]

# Response to Reviewers (Side-by-Side Format)

Manuscript Title: Latent Tuberculosis Infection in Focus: Evaluating Public Concerns, Treatment Perceptions, and Awareness in King Sabata Dalindyebo Local Municipality, Eastern Cape, South Africa

Journal: PLOS ONE

Revision Type: Major Revision

We thank the reviewers for their constructive feedback. The table below outlines the reviewer comments and corresponding author responses and revisions made.

| Reviewer Comment | Author Response and Revisions Made |
| --- | --- |
| Other statistical model analyses can be explored to check for factors affecting knowledge and attitude, controlling for confounders. | A multivariate logistic regression analysis (Table 6) was added to identify independent predictors of LTBI knowledge and attitude. This model adjusted for confounders (age, gender, education, and prior TB contact). Model fit statistics (Nagelkerke R², Hosmer–Lemeshow) were included. |
| Tables are missing with the manuscript submission. | All referenced tables (Tables 2–6) were inserted. A comprehensive Data Availability Statement was added, confirming that the dataset is publicly available via an open-access repository (e.g., Figshare or Zenodo). |
| The PLOS Data policy requires all data underlying findings to be fully available. | A revised Data Availability Statement specifies the dataset location and access details. All data underlying means, medians, and variance measures are available without restriction, following PLOS ONE guidelines. |
| Some sentences are ambiguous and can be improved. | A thorough language edit was conducted to improve clarity, precision, and structure. Ambiguous phrases were revised, transitions clarified, and all grammatical issues corrected. |
| The discussion should more clearly link findings to existing literature and implications. | The Discussion section was substantially expanded. The section now integrates global and local studies, highlighting behavioral, structural, and policy implications consistent with WHO’s End TB Strategy. |
| Strengthen interpretation by including visual or conceptual linkage between key variables. | A new figure (Figure 2: Predictive Pathway for Improved LTBI Outcomes) was created to visually illustrate relationships among barriers, perceptions, and intervention outcomes. |
| Clarify ethical and methodological standards. | The ethics section was updated to include explicit details on participant consent, confidentiality, and adherence to institutional and national ethical standards. |
| The abstract should be concise and follow PLOS ONE structure. | The Abstract was rewritten using the standard PLOS ONE structure (Background, Methods, Results, Conclusion). It now includes key quantitative findings and clearer policy relevance. |

## Summary of Major Improvements

• Inclusion of multivariate logistic regression model (Table 6)
• Addition of all missing tables and figures
• Strengthened data availability and repository compliance
• Language and clarity improvements throughout
• Expanded discussion linking findings to global evidence
• Addition of conceptual pathway figure
• Clarified ethical considerations
• Revised abstract aligned with PLOS ONE format

These revisions fully address the reviewers’ feedback and enhance the manuscript’s methodological rigor, clarity, and compliance with PLOS ONE publication standards.
