# Supplementary material for: Latent tuberculosis infection in focus: Evaluating public concerns, treatment perceptions, and awareness in King Sabatha Dalindyebo Local Municipality, Eastern Cape, South Africa
Source: PLOS Glob Public Health. 2026 Sep 15;6(9):e0005130. doi: 10.1371/journal.pgph.0005130 (PMC13577523; doi:10.1371/journal.pgph.0005130)
Supplement: Supplementary file 4 [file pgph.0005130.s004.docx]

The statistical methodology of this cross-sectional, community-based survey contains significant deficiencies, necessitating a **MAJOR REVISION**. Although the authors included 245 participants, they failed to provide any explanation regarding the **sample size and sample power calculation** to demonstrate that their findings have sufficient statistical power. Furthermore, while parametric tests were used, there is a crucial omission of **any information or testing regarding the data's conformity to a normal distribution**. This raises serious questions about the validity of the chosen statistical analyses and the reliability of the results. Lastly, the lack of **dedicated tables for statistical analysis results**, with findings only highlighted in the text, is unacceptable for academic reporting standards as it prevents a complete and transparent evaluation of the study's quantitative findings. All these methodological shortcomings must be addressed and completed before the study can be reconsidered.

**Citations for the above response:**

1. "...245 individuals were included. However, was the sample size and sample power calculated before including these 245 individuals? I did not see this."
2. "Perhaps this study, with 245 participants, was insufficient to calculate the sample size."
3. "There is no information regarding the data's conformity to a normal distribution. Parametric tests were used, however."
4. "There are no tables for the statistical analysis results. Only the text highlights whether the results are statistically significant or insignificant."
5. "Decision: The study must complete all the statistical incompleteness requested above.
