# Supplementary material for: Latent tuberculosis infection in focus: Evaluating public concerns, treatment perceptions, and awareness in King Sabatha Dalindyebo Local Municipality, Eastern Cape, South Africa
Source: PLOS Glob Public Health. 2026 Sep 15;6(9):e0005130. doi: 10.1371/journal.pgph.0005130 (PMC13577523; doi:10.1371/journal.pgph.0005130)
Supplement: Supplementary file 5 [file pgph.0005130.s005.docx]

PGPH-D-25-02258R1
Latent Tuberculosis Infection in Focus: Evaluating Public Concerns, Treatment Perceptions, and Awareness in King Sabatha Dalindyebo Local Municipality, Eastern Cape, South Africa
PLOS Global Public Health (Global Plos One)

Reviewer 2

Reviewers Comments

1. "...245 individuals were included. However, was the sample size and sample power calculated before including these 245 individuals? I did not see this."

Response:
Thank you for this important comment. This study was an exploratory, cross-sectional, community-based survey that utilized a pragmatic sampling approach within the King Sabata Dalindyebo Local Municipality. A formal a priori sample size or statistical power calculation was not performed because there was no prior local data on LTBI awareness and perceptions in this setting. Instead, the sample size (n = 245) was determined based on feasibility, resource availability, and the study period. We acknowledge that the lack of formal power calculation is a limitation, and this has now been clearly stated in the revised manuscript. Future studies will incorporate formal sample-size estimation based on the effect sizes observed in this study.

Comments:

2. "Perhaps this study, with 245 participants, was insufficient to calculate the sample size."

Response:

Thank you for this insightful comment. We agree that the relatively modest sample size (n = 245) may limit the precision of estimates and the ability to detect smaller effect sizes. This study was designed as an exploratory, cross-sectional assessment to generate baseline data on LTBI awareness, perceptions, and barriers in a previously under-researched rural setting. A formal a priori sample size or power calculation was not conducted, as there were limited prior data to inform parameter assumptions for such calculations in this context. The achieved sample size reflects pragmatic considerations, including feasibility, available resources, and the study timeframe. We acknowledge this as a limitation and have now clarified it in the revised manuscript. Future studies with larger, adequately powered samples are recommended to validate and extend these findings.

Comments:

3. "There is no information regarding the data's conformity to a normal distribution. Parametric tests were used, however."

Response: Thank you for this important observation. We agree that assessing the distribution of continuous variables is essential for selecting appropriate statistical tests. In the revised manuscript, we have clarified that the distribution of continuous variables was assessed using both visual (histograms and Q–Q plots) and statistical methods (e.g., Shapiro–Wilk test). Based on these assessments, most continuous variables were not normally distributed; therefore, non-parametric tests (Mann–Whitney U test) were primarily used for group comparisons. Where appropriate, parametric tests were applied only when assumptions of normality were reasonably met. We have revised the Methods section to clearly reflect this approach and ensure transparency in the statistical analysis.

Comments:

4. "There are no tables for the statistical analysis results. Only the text highlights whether the results are statistically significant or insignificant."

Response:

Thank you for this important observation. We agree that presenting statistical results in tabular format improves clarity, transparency, and interpretability. In response, we have revised the manuscript to include detailed tables summarizing the results of the statistical analyses. Specifically:

- **Table 2** now presents the univariable logistic regression results, including odds ratios (OR), 95% confidence intervals (CI), and p-values.
- **Table 3** presents the fully adjusted multivariable models (logistic regression and Poisson regression), including adjusted estimates and confidence intervals.
- **Table 4** presents the reduced multivariable model.

These additions ensure that all statistical findings are clearly presented and allow readers to fully evaluate the magnitude, direction, and precision of the observed associations.

Comments:

5. "Decision: The study must complete all the statistical incompleteness requested above.

Response:

Thank you for this important summary comment. We have carefully addressed all concerns related to statistical completeness and transparency throughout the manuscript.

Specifically, the following revisions have been made:

1. **Assessment of data distribution:**
   We have clarified that the distribution of continuous variables was assessed using visual methods (histograms, Q–Q plots) and statistical tests (Shapiro–Wilk test), and that appropriate nonparametric tests were applied when normality assumptions were not met.
2. **Appropriate statistical test selection:**
   The Methods section has been revised to clearly justify the use of both nonparametric and parametric tests, ensuring alignment with the data characteristics.
3. **Comprehensive tabular presentation of results:**
   Detailed statistical results are now presented in:
   - **Table 2:** Univariable analysis
   - **Table 3:** Fully adjusted multivariable models
   - **Table 4:** Reduced multivariable model

These tables include effect estimates (OR/aRR), 95% confidence intervals, and p-values.

4. **Improved reporting consistency:**
   All results are now consistently reported using standard epidemiological formats, including median (IQR) for continuous variables and n (%) for categorical variables.
5. **Clarification of modelling approach:**
   The rationale for multivariable modelling, variable selection, and handling of confounders has been explicitly described.
6. **Transparent reporting of limitations:**
   Limitations related to sample size, statistical power, and observational design have been clearly acknowledged.

These revisions ensure that the statistical analysis is complete, transparent, and aligned with standard epidemiological reporting guidelines.
