# Supplementary material for: Latent tuberculosis infection in focus: Evaluating public concerns, treatment perceptions, and awareness in King Sabatha Dalindyebo Local Municipality, Eastern Cape, South Africa
Source: PLOS Glob Public Health. 2026 Sep 15;6(9):e0005130. doi: 10.1371/journal.pgph.0005130 (PMC13577523; doi:10.1371/journal.pgph.0005130)
Supplement: Supplementary file 6 [file pgph.0005130.s006.docx]

**Response to Reviewer 2**

We sincerely thank Reviewer 2 for the careful evaluation of our manuscript and for the constructive comments and recommendations. We appreciate the insightful feedback, which has significantly improved the clarity, methodological transparency, and overall scientific quality of the manuscript. We have carefully considered all comments and revised the manuscript accordingly. Our detailed responses are provided below.

**Comment 1: Study Rationale and Background**

**Reviewer Comment:**
The introduction requires additional context regarding the importance of latent tuberculosis infection (LTBI) and its relevance to tuberculosis prevention strategies in South Africa.

**Response:**

We thank the reviewer for this important suggestion. The Introduction has been substantially revised to provide a more comprehensive overview of the epidemiology and public health significance of LTBI globally and within South Africa. We also expanded the discussion of LTBI screening, preventive treatment, and its role within national and global tuberculosis elimination strategies, including alignment with the World Health Organization End TB Strategy.

**Changes Made:**

- Expanded the Introduction section.
- Added recent literature and policy context related to LTBI prevention and control.
- Clarified the rationale and public health significance of the study.

**Comment 2: Sampling and Participant Recruitment**

**Reviewer Comment:**
Additional information regarding participant recruitment and sampling procedures is required.

**Response:**

We appreciate this observation. The Methods section has been revised to provide a detailed description of participant recruitment procedures, inclusion criteria, exclusion criteria, community outreach activities, and the rationale for the exploratory community-based sampling approach. We further clarified why probability-based sampling was not feasible within the study setting.

**Changes Made:**

- Expanded the Sampling and Participants section.
- Added justification for the exploratory study design.
- Clarified recruitment procedures and study setting.

**Comment 3: Sample Size and Statistical Power**

**Reviewer Comment:**
Please clarify whether a sample size calculation or statistical power analysis was conducted.

**Response:**

We thank the reviewer for highlighting this important methodological issue. We have now clarified that this exploratory study did not include a formal a priori sample size calculation because limited local data were available to inform assumptions regarding expected effect sizes and outcome variability. We further acknowledge in the Limitations section that the sample size may have limited statistical power to detect smaller associations and that findings should therefore be interpreted as exploratory and hypothesis-generating.

**Changes Made:**

- Revised the Sampling and Participants section.
- Expanded the Limitations section.

**Comment 4: Statistical Analysis**

**Reviewer Comment:**
Please provide additional details regarding statistical methods and assumptions.

**Response:**

We agree and have substantially expanded the Data Analysis section. The revised manuscript now describes assessment of data distribution using histograms, Q–Q plots, and the Shapiro–Wilk test. We also clarify the selection of parametric and non-parametric statistical tests, reporting of confidence intervals, and procedures used for multivariable logistic regression analyses.

**Changes Made:**

- Expanded Data Analysis section.
- Added information regarding normality assessment and model diagnostics.
- Clarified reporting of effect estimates and confidence intervals.

**Comment 5: Presentation of Results**

**Reviewer Comment:**
Statistical findings should be presented more comprehensively.

**Response:**

We appreciate this recommendation. The Results section has been revised to improve clarity and transparency. Additional statistical information, including effect estimates, confidence intervals, and p-values, has been incorporated into the tables and accompanying narrative descriptions. Tables and figure legends have also been revised to improve interpretability.

**Changes Made:**

- Revised Tables 2–6.
- Expanded interpretation of key findings.
- Updated figure captions and explanatory notes.

**Comment 6: Interpretation of Findings**

**Reviewer Comment:**
The discussion should avoid causal language given the study design.

**Response:**

We agree completely. Throughout the manuscript, causal language has been removed and replaced with language that appropriately reflects associations observed within a cross-sectional observational study. The Discussion, Conclusion, and figure descriptions have been revised accordingly.

**Changes Made:**

- Revised Discussion section.
- Revised Conclusion section.
- Revised conceptual framework descriptions and figure captions.

**Comment 7: Conceptual Framework**

**Reviewer Comment:**
The framework appears predictive and may imply causal relationships.

**Response:**

We appreciate this important observation. The figure has been revised and renamed as a conceptual framework informed by study findings and existing literature. The accompanying text now explicitly states that the framework is intended for conceptual and programmatic purposes and should not be interpreted as a causal or empirically validated model.

**Changes Made:**

- Revised Figure 2.
- Updated figure caption.
- Revised Discussion section.

**Comment 8: Limitations**

**Reviewer Comment:**
The limitations require further discussion.

**Response:**

We agree and have substantially expanded the Limitations section. The revised manuscript now discusses the implications of the cross-sectional design, absence of a formal sample size calculation, potential limitations in statistical power, self-reported data, possible recall and social desirability bias, limited generalizability, and the conceptual nature of the proposed framework.

**Changes Made:**

- Expanded Limitations section.

**Comment 9: Language and Clarity**

**Reviewer Comment:**
The manuscript would benefit from improved clarity and organization.

**Response:**

We thank the reviewer for this suggestion. The manuscript has undergone extensive editing to improve clarity, readability, consistency of terminology, and overall flow. Several sections were reorganized, redundant text was removed, and abbreviations were standardized throughout the manuscript.

**Changes Made:**

- Comprehensive language editing.
- Improved manuscript structure and flow.
- Standardized abbreviations throughout the manuscript.

**Closing Statement**

We sincerely thank Reviewer 2 for the thoughtful and constructive comments. We believe that the revisions undertaken have substantially strengthened the manuscript and improved its methodological transparency, scientific rigor, and clarity.
