# Supplementary material for: Latent tuberculosis infection in focus: Evaluating public concerns, treatment perceptions, and awareness in King Sabatha Dalindyebo Local Municipality, Eastern Cape, South Africa
Source: PLOS Glob Public Health. 2026 Sep 15;6(9):e0005130. doi: 10.1371/journal.pgph.0005130 (PMC13577523; doi:10.1371/journal.pgph.0005130)
Supplement: Peer review history

# Peer review: Decision Letter 0

**DOI:** 10.1371/journal.pgph.0005130.r001

**Authors:** Isdore Chola Shamputa

29 Sep 2025

PGPH-D-25-02258

Latent Tuberculosis Infection in Focus: Evaluating Public Concerns, Treatment Perceptions, and Awareness in King Sabatha Dalindyebo Local Municipality, Eastern Cape, South Africa

PLOS Global Public Health

Dear Dr. Dlatu,

Thank you for submitting your manuscript to PLOS Global Public Health. After careful consideration, we feel that it has merit but does not fully meet PLOS Global Public Health’s publication criteria as it currently stands. Therefore, we invite you to submit a revised version of the manuscript that addresses the points raised during the review process.

Please submit your revised manuscript by 26 October 2025. If you will need more time than this to complete your revisions, please reply to this message or contact the journal office at globalpubhealth@plos.org. When you're ready to submit your revision, log on to https://www.editorialmanager.com/pgph/ and select the 'Submissions Needing Revision' folder to locate your manuscript file.

Please include the following items when submitting your revised manuscript:

- A rebuttal letter that responds to each point raised by the editor and reviewer(s). You should upload this letter as a separate file labeled 'Response to Reviewers'.
- A marked-up copy of your manuscript that highlights changes made to the original version. You should upload this as a separate file labeled 'Revised Manuscript with Track Changes'.
- An unmarked version of your revised paper without tracked changes. You should upload this as a separate file labeled 'Manuscript'.

Guidelines for resubmitting your figure files are available below the reviewer comments at the end of this letter.

We look forward to receiving your revised manuscript.

Kind regards,

Isdore Chola Shamputa, PhD

Academic Editor

PLOS Global Public Health

Journal Requirements:

If the reviewer comments include a recommendation to cite specific previously published works, please review and evaluate these publications to determine whether they are relevant and should be cited. There is no requirement to cite these works unless the editor has indicated otherwise.

Additional Editor Comments (if provided):

Two reviewers have provided their feedback on your manuscript and have requested additional information and clarification on several points. Please review and address their comments, and submit a revised version of the manuscript with your responses for further consideration at your earliest convenience..

[Note: HTML markup is below. Please do not edit.]

Reviewers' comments:

Reviewer’s Responses to Questions

**Comments to the Author**

1. Does this manuscript meet PLOS Global Public Health’s publication criteria?

Reviewer #1: Yes

Reviewer #2: Yes

**********

2. Has the statistical analysis been performed appropriately and rigorously?

Reviewer #1: Yes

Reviewer #2: No

**********

3. Have the authors made all data underlying the findings in their manuscript fully available (please refer to the Data Availability Statement at the start of the manuscript PDF file)?

The PLOS Data policy

Reviewer #1: Yes

Reviewer #2: Yes

**********

4. Is the manuscript presented in an intelligible fashion and written in standard English?

Reviewer #1: Yes

Reviewer #2: Yes

**********

Reviewer #1: The manuscript is fairly well written. But there are some ambiguous sentences which is confusing and can be improved. Please attach the tables on resubmission, as it is difficult to look at results without it

Reviewer #2: This was a cross-sectional, community-based survey conducted in the KSD Local Municipality, located in the Oliver Reginald (O.R.) Tambo District of the Eastern Cape Province. The study was conducted between September 10 and November 2024.

The study was conducted following the Declaration of Helsinki and approved by the Research Ethics and Biosafety Committee of the Faculty of Medicine and Health Sciences of Walter Sisulu University (ref. no. 084/2024) and the Eastern Cape Department of Health (ref. no. EC_202409_008).

The study included individuals aged 18 and over, and 245 individuals were included. However, was the sample size and sample power calculated before including these 245 individuals? I did not see this. Perhaps this study, with 245 participants, was insufficient to calculate the sample size. The author should provide a detailed explanation of the sample selection.

There is no information regarding the data’s conformity to normal distribution. Parametric tests were used, however.

The figures used in the study are sufficient.

There are no tables for the statistical analysis results. Only the text highlights whether the results are statistically significant or insignificant.

Decision: The study must complete all the statistically incompleteness requested above.

MAJOR REVISION

**********

what does this mean?). If published, this will include your full peer review and any attached files.

**Do you want your identity to be public for this peer review?**  If you choose “no”, your identity will remain anonymous but your review may still be made public.

For information about this choice, including consent withdrawal, please see our Privacy Policy

Reviewer #1: No

Reviewer #2: No

**********

[NOTE: If reviewer comments were submitted as an attachment file, they will be attached to this email and accessible via the submission site. Please log into your account, locate the manuscript record, and check for the action link "View Attachments". If this link does not appear, there are no attachment files.]

Attachment Submitted filename: comments.docx

Attachment Submitted filename: Paper.docx

# Author comment: Author response to Decision Letter 1

**DOI:** 10.1371/journal.pgph.0005130.r002

10 Nov 2025

Attachment Submitted filename: 10 Nov 2025 Response_to_Reviewers_Side_by_Side_PLOS_ONE.docx

# Peer review: Decision Letter 1

**DOI:** 10.1371/journal.pgph.0005130.r003

**Authors:** Helen Howard

15 Feb 2026

PGPH-D-25-02258R1

Latent Tuberculosis Infection in Focus: Evaluating Public Concerns, Treatment Perceptions, and Awareness in King Sabatha Dalindyebo Local Municipality, Eastern Cape, South Africa

PLOS Global Public Health

Dear Dr. Dlatu,

Thank you for submitting your manuscript to PLOS Global Public Health. After careful consideration, we feel that it has merit but does not fully meet PLOS Global Public Health’s publication criteria as it currently stands. Therefore, we invite you to submit a revised version of the manuscript that addresses the points raised during the review process.

Please submit your revised manuscript by Mar 30 2026 11:59PM. If you will need more time than this to complete your revisions, please reply to this message or contact the journal office at globalpubhealth@plos.org. When you're ready to submit your revision, log on to https://www.editorialmanager.com/pgph/ and select the 'Submissions Needing Revision' folder to locate your manuscript file.

Please include the following items when submitting your revised manuscript:

- A letter that responds to each point raised by the editor and reviewer(s). You should upload this letter as a separate file labeled 'Response to Reviewers'.
- A marked-up copy of your manuscript that highlights changes made to the original version. You should upload this as a separate file labeled 'Revised Manuscript with Track Changes'.
- An unmarked version of your revised paper without tracked changes. You should upload this as a separate file labeled 'Manuscript'.

Guidelines for resubmitting your figure files are available below the reviewer comments at the end of this letter.

We look forward to receiving your revised manuscript.

Kind regards,

Helen Howard

Staff Editor

PLOS Global Public Health

Journal Requirements:

If the reviewer comments include a recommendation to cite specific previously published works, please review and evaluate these publications to determine whether they are relevant and should be cited. There is no requirement to cite these works unless the editor has indicated otherwise.

Additional Editor Comments (if provided):

[Note: HTML markup is below. Please do not edit.]

Reviewers' comments:

Reviewer’s Responses to Questions

**Comments to the Author**

Reviewer #2: All comments have been addressed

**********

publication criteria?

Reviewer #2: Partly

**********

3. Has the statistical analysis been performed appropriately and rigorously?

Reviewer #2: No

**********

4. Have the authors made all data underlying the findings in their manuscript fully available (please refer to the Data Availability Statement at the start of the manuscript PDF file)?

The PLOS Data policy

Reviewer #2: No

**********

5. Is the manuscript presented in an intelligible fashion and written in standard English?

Reviewer #2: Yes

**********

Reviewer #2: The statistical methodology of this cross-sectional, community-based survey contains significant deficiencies, necessitating a MAJOR REVISION. Although the authors included 245 participants, they failed to provide any explanation regarding the sample size and sample power calculation to demonstrate that their findings have sufficient statistical power. Furthermore, while parametric tests were used, there is a crucial omission of any information or testing regarding the data’s conformity to a normal distribution. This raises serious questions about the validity of the chosen statistical analyses and the reliability of the results. Lastly, the lack of dedicated tables for statistical analysis results, with findings only highlighted in the text, is unacceptable for academic reporting standards as it prevents a complete and transparent evaluation of the study’s quantitative findings. All these methodological shortcomings must be addressed and completed before the study can be reconsidered.

________________________________________

Citations for the above response:

1. "...245 individuals were included. However, was the sample size and sample power calculated before including these 245 individuals? I did not see this."

2. "Perhaps this study, with 245 participants, was insufficient to calculate the sample size."

3. "There is no information regarding the data’s conformity to a normal distribution. Parametric tests were used, however."

4. "There are no tables for the statistical analysis results. Only the text highlights whether the results are statistically significant or insignificant."

5. "Decision: The study must complete all the statistical incompleteness requested above.

**********

what does this mean?). If published, this will include your full peer review and any attached files.

**Do you want your identity to be public for this peer review?**  If you choose “no”, your identity will remain anonymous but your review may still be made public.

For information about this choice, including consent withdrawal, please see our Privacy Policy

Reviewer #2: No

**********

[NOTE: If reviewer comments were submitted as an attachment file, they will be attached to this email and accessible via the submission site. Please log into your account, locate the manuscript record, and check for the action link "View Attachments". If this link does not appear, there are no attachment files.]

Attachment Submitted filename: Review.docx

# Author comment: Author response to Decision Letter 2

**DOI:** 10.1371/journal.pgph.0005130.r004

20 Apr 2026

Attachment Submitted filename: 19 April 2026 Global Plos One Reviewers Comments.docx

# Peer review: Decision Letter 2

**DOI:** 10.1371/journal.pgph.0005130.r005

**Authors:** Helen Howard

2 Jun 2026

PGPH-D-25-02258R2

Latent Tuberculosis Infection in Focus: Evaluating Public Concerns, Treatment Perceptions, and Awareness in King Sabatha Dalindyebo Local Municipality, Eastern Cape, South Africa

PLOS Global Public Health

Dear Dr. Dlatu,

Thank you for submitting your manuscript to PLOS Global Public Health. After careful consideration, we feel that it has merit but does not fully meet PLOS Global Public Health’s publication criteria as it currently stands. Therefore, we invite you to submit a revised version of the manuscript that addresses the points raised during the review process.

In particular, we note that the original comments provided by reviewer 1 do not appear to have been responded to nor fully addressed in the manuscript. Could you please reconsider the previous comments provided by reviewer 1 - as provided in the attachment "paper with comments".

Please submit your revised manuscript by Jul 31 2026 11:59PM. If you will need more time than this to complete your revisions, please reply to this message or contact the journal office at globalpubhealth@plos.org. When you're ready to submit your revision, log on to https://www.editorialmanager.com/pgph/ and select the 'Submissions Needing Revision' folder to locate your manuscript file.

Please include the following items when submitting your revised manuscript:

- A letter that responds to each point raised by the editor and reviewer(s). You should upload this letter as a separate file labeled 'Response to Reviewers'.
- A marked-up copy of your manuscript that highlights changes made to the original version. You should upload this as a separate file labeled 'Revised Manuscript with Track Changes'.
- An unmarked version of your revised paper without tracked changes. You should upload this as a separate file labeled 'Manuscript'.

Guidelines for resubmitting your figure files are available below the reviewer comments at the end of this letter.

As the corresponding author, your ORCID iD is verified in the submission system and will appear in the published article. PLOS supports the use of ORCID, and we encourage all coauthors to register for an ORCID iD and use it as well. Please encourage your coauthors to verify their ORCID iD within the submission system before final acceptance, as unverified ORCID iDs will not appear in the published article. *Only*  the individual author can complete the verification step; PLOS staff *cannot*  verify ORCID iDs on behalf of authors.

We look forward to receiving your revised manuscript.

Kind regards,

Helen Howard

Staff Editor

PLOS Global Public Health

Journal Requirements:

If the reviewer comments include a recommendation to cite specific previously published works, please review and evaluate these publications to determine whether they are relevant and should be cited. There is no requirement to cite these works unless the editor has indicated otherwise.

Additional Editor Comments (if provided):

[Note: HTML markup is below. Please do not edit.]

Reviewers' comments:

Reviewer’s Responses to Questions

**Comments to the Author**

Reviewer #2: All comments have been addressed

**********

publication criteria?

Reviewer #2: Yes

**********

3. Has the statistical analysis been performed appropriately and rigorously?

Reviewer #2: Yes

**********

4. Have the authors made all data underlying the findings in their manuscript fully available (please refer to the Data Availability Statement at the start of the manuscript PDF file)?

The PLOS Data policy

Reviewer #2: Yes

**********

5. Is the manuscript presented in an intelligible fashion and written in standard English?

Reviewer #2: Yes

**********

Reviewer #2: Sample Size and Power Calculation

1. "...245 individuals were included. However, was the sample size and sample power calculated before including these 245 individuals? I did not see this."

Failure to provide an explanation regarding sample size and sample power calculation for the 245 participants.

Author’s Correction: The author has acknowledged this as a limitation. In the revised manuscript, they clarified that while a formal a priori power calculation was not initially detailed, the limitations related to sample size and statistical power are now explicitly discussed in the "Limitations" section to ensure transparency regarding the study’s scope.

Data Conformity and Normality Testing

2. "There is no information regarding the data’s conformity to a normal distribution. Parametric tests were used, however."

Use of parametric tests without information on whether the data followed a normal distribution.

Author’s Correction: The author updated the "Methods" section to state that the distribution of continuous variables was assessed using the Shapiro–Wilk test. They clarified that non-parametric tests were applied in cases where normality assumptions were not met, justifying the choice of statistical tests based on the ata characteristics.

Statistical Analysis Tables

3. "There are no tables for the statistical analysis results. Only the text highlights whether the results are statistically significant or insignificant."

Lack of dedicated tables for statistical analysis (results only highlighted in text).

Author’s Correction: This has been fully rectified. The author has added comprehensive tables to present the quantitative findings:

Table 2: Univariable analysis.

Table 3: Fully adjusted multivariable models.

Table 4: Reduced multivariable model. These tables now include effect estimates (OR/aRR), 95% confidence intervals, and p-values, meeting the standard for academic reporting.

4. General Reporting Standards

Author’s Correction: The author has adopted standard epidemiological formats, such as using median (IQR) for continuous variables and n (%) for categorical variables, ensuring a more transparent and professional evaluation of the study’s findings.

Conclusion: The author has systematically addressed the reviewer’s critiques by adding the missing statistical tests, providing the required data tables, and acknowledging the power calculation limitations.

Best regards

**********

what does this mean?). If published, this will include your full peer review and any attached files.

**Do you want your identity to be public for this peer review?**  If you choose “no”, your identity will remain anonymous but your review may still be made public.

For information about this choice, including consent withdrawal, please see our Privacy Policy

Reviewer #2: No

**********

[NOTE: If reviewer comments were submitted as an attachment file, they will be attached to this email and accessible via the submission site. Please log into your account, locate the manuscript record, and check for the action link "View Attachments". If this link does not appear, there are no attachment files.]

# Author comment: Author response to Decision Letter 3

**DOI:** 10.1371/journal.pgph.0005130.r006

22 Jun 2026

Attachment Submitted filename: 22 June Global Plos One Response to Reviewer 2.docx

# Editor report: Decision Letter 3

**DOI:** 10.1371/journal.pgph.0005130.r007

**Authors:** Julia Robinson

7 Jul 2026

Latent Tuberculosis Infection in Focus: Evaluating Public Concerns, Treatment Perceptions, and Awareness in King Sabatha Dalindyebo Local Municipality, Eastern Cape, South Africa

PGPH-D-25-02258R3

Dear Dr Dlatu,

We are pleased to inform you that your manuscript 'Latent Tuberculosis Infection in Focus: Evaluating Public Concerns, Treatment Perceptions, and Awareness in King Sabatha Dalindyebo Local Municipality, Eastern Cape, South Africa' has been provisionally accepted for publication in PLOS Global Public Health.

Before your manuscript can be formally accepted you will need to complete some formatting changes, which you will receive in a follow up email. A member of our team will be in touch with a set of requests.

Please note that your manuscript will not be scheduled for publication until you have made the required changes, so a swift response is appreciated.

IMPORTANT: The editorial review process is now complete. PLOS will only permit corrections to spelling, formatting or significant scientific errors from this point onwards. Requests for major changes, or any which affect the scientific understanding of your work, will cause delays to the publication date of your manuscript.

If your institution or institutions have a press office, please notify them about your upcoming paper to help maximize its impact. If they'll be preparing press materials, please inform our press team as soon as possible -- no later than 48 hours after receiving the formal acceptance. Your manuscript will remain under strict press embargo until 2 pm Eastern Time on the date of publication. For more information, please contact globalpubhealth@plos.org.

Thank you again for supporting Open Access publishing; we are looking forward to publishing your work in PLOS Global Public Health.

Best regards,

Julia Robinson

Executive Editor

PLOS Global Public Health

***********************************************************

Reviewer Comments (if any, and for reference):
